# Nurse performance in hospital financial auditing: a single case study

**DOI:** 10.1590/1980-220X-REEUSP-2024-0391en

**Published:** 2025-07-07

**Authors:** Silvia Helena Meneguin, Vânia dos Santos Nunes Nogueira, Silvia Cristina Mangini Bocchi

**Affiliations:** 1 Universidade Estadual Paulista, Faculdade de Medicina de Botucatu, Botucatu, SP, Brazil; 2 Universidade Estadual Paulista, Faculdade de Medicina de Botucatu, Departamento de Clínica Médica, Botucatu, SP, Brazil.; 3 Universidade Estadual Paulista, Faculdade de Medicina de Botucatu, Departamento de Enfermagem, Botucatu, SP, Brazil.

**Keywords:** Nurse’s Role, Nursing Audit, Supplemental Health, Financial Management, Health Care Economics and Organizations

## Abstract

**Objective:**

To analyze the performance of nurses in retrospective financial audits, compared with that of “non-nurses”, for remuneration of a hospital by supplemental health insurance providers.

**Method:**

A single, quantitative, retrospective, documentary and analytical case study, using as the unit of analysis a set of 238 accounting bills invoiced by “non-nurses” and audited by nurses for outpatient procedures in the hemodynamics service of a public hospital in the state of São Paulo, from 2019 to 2021.

**Results:**

Underbilling was detected in 100% of the billing groups in the “billed < audited” category, regardless of the procedures performed, with a cumulative total of R$ 1,024,692.00, which, updated to September 2024, would correspond to R$ 1,344,125.00. In cases of undue charges, “billed > audited”, there was a cumulative total of R$ 647,828.00.

**Conclusion:**

Retrospective financial audits performed by nurses increase hospital revenue. Finally, this study contributes by demonstrating the need to train these professionals in economic and financial skills in health care, highlighting the importance of the emerging role of financial auditor nurses in the health care market.

## INTRODUCTION

Faced with the increased risk of financial and economic unsustainability of their businesses due to the rapid increase in health care costs^(1)^, the health care market has mobilized to manage reduced budgets so as to balance costs, expenditures, and revenues in order to ensure the survival of their organizations.

The scarcity of financial resources is the result of technological advances and demographic change, accompanied by an epidemiological transition to an ageing population^(2)^. This context highlights the need for health care managers who are able to provide an effective cost management system without compromising care quality, with the ultimate goal of ensuring the efficient distribution of resources^(3)^.

Nursing care is known to consume approximately one third of hospital budgets^(4)^, and it requires nurses with knowledge and skills in decision making regarding institutional structure, processes, and outcomes. In this context, it is necessary to train nurses in cost management as another tool to be used in decision-making processes for the management of nursing services^(3)^.

In this regard, the literature has corroborated the need for nurses who aspire to the position of or work as financial managers in health care institutions to acquire economic and financial competencies^(5,(6,7)^, so that they can develop actions to ensure the sustainability of the care provided by these organizations^(8)^, demonstrating that this is a promising area of performance for professionals in the role of auditors^(9)^.

This study used a retrospective post-discharge audit, performed by systematically reviewing hospital service charges based on patient records^(10)^ to assess administrative and care-related distortions in a hemodynamics service.

There are few scientific articles linking health economics with the implementation of internal financial audits by nurses^(8,11,12)^.

In light of the above, the question for this study was: How can nurses’ performance in retrospective financial audits of hospital accounts billed by “non-nurses” contribute to hospital revenue?

The hypothesis was that adopting a process of prior review of hospital accounts by nurses would increase the effectiveness of billing management.

To test this hypothesis, the objective was defined as follows: to analyze the performance of nurses in retrospective financial auditing compared to that performed by “non-nurses” in the remuneration of a hospital by health insurance companies (HICs).

## METHOD

### Study Design

This is a quantitative, retrospective, documentary and analytical single-case study^(13)^. The unit of analysis was the economic evaluation of health care, and therefore, the CHEERS roadmap was used to report it^(14)^.

### Setting and unit of Analysis

The unit of analysis consisted of a set of 238 billed accounts using the Fee-for-Service payment model (open account for billing items)^(15)^ from January 1, 2019, to December 31, 2021, billed by “non-nurses” and audited by nurses. These accounts were from beneficiaries of seven HICs who underwent diagnostic and therapeutic procedures at the hemodynamics outpatient service of a public hospital in the state of São Paulo, Brazil.

This set was selected from 631 billed accounts, excluding 393 whose beneficiaries were hospitalized or came from other payment models. The analysis was performed from the hospital’s perspective and from the perspective of the financial impact as audited by nurses.

It should be noted that the location and time period for this study were chosen because of the costly nature of diagnostic and therapeutic procedures and the lack of control associated with them prior to the implementation of retrospective auditing of accounts by nurses. From January 1, 2019, to December 31, 2021, these accounts were billed by administrative professionals with no health care training and, therefore, they are referred to in this study as “non-nurse” employees.

The hemodynamics service is part of the hospital’s Diagnostic and Therapeutic Procedures Center, with an average of 3,700 exams being performed per month, mostly for beneficiaries of the Unified Health System (SUS), whereas only 6% of such exams are covered by private health insurance. The profits from this service are used to improve the quality of the care provided by the institution.

### Billing Process and Account Auditing

The accounting of the services provided by the Hemodynamics Department is divided into billing groups (BGs), including room charges; gas therapy; equipment; materials; medications; orthoses, prostheses and special materials (OPSMs); and medical fees.

The pricing of daily rates and fees follows the service agreements between the insurance companies and the hospital, as well as the reference tables in the health care market, Simpro^(16)^ and Brasíndice^(17)^, for the remuneration of materials and medicines. For OPSMs, however, the prices quoted by the supplier are used.

The billing process begins with the beneficiary’s admission to the service and the request for authorization from the HICs, according to the pre- and post-procedure needs, and ends only when a payment invoice is sent to the insurers, after being checked by a nurse specialized in this area.

The purpose of this audit is to review the account in accordance with the service agreement signed with the HIC, the multidisciplinary care records in the beneficiary’s electronic medical record, and the authorizations relevant to the entire care provision process. This process allows for adjustments as needed to include or exclude items from the account so that it can be sent to the HIC with the accuracy and integrity of a quality service.

The post-billing process involves several departments, as it is linked to the payment and remittance of physician fees associated with the outcome of appeals for disallowances to be removed from the hospital bill.

### Variables

*Characterizing the beneficiaries at the unit of analysis:* gender (F/M); age groups in years (20 to 29, 30 to 39, 40 to 49, 50 to 59, and ≥ 60); medical diagnoses in test requests (classified by groups and chapters of the International Classification of Diseases - ICD-10)^(18)^; classification of diagnostic and therapeutic procedures (angiography, cardiac catheterization, angioplasty with cardiac and cerebral stents, surgical interatrial correction, chemoembolization, and transcatheter valve implantation); access route for tests (brachial, radial, and femoral).

*Independent:* the types of procedures, grouped into therapeutic and diagnostic, performed in the hemodynamics service.

*Dependent:* results between invoices issued by administrative “non-nurse” staff (and not from the health care field) and audited by nurses with audit training of items classified in the distribution ranges “billed = audited”, “billed < audited”, and “billed > audited”, for this study, in the BGs referred to as rates: room, equipment, medications, materials, OPSMs, and gas therapy. The efficiency of the nurse auditor was considered if he/she detected items in the “billed < audited” range, since the items grouped in the “billed > audited” range are mostly disallowed.

### Ethical, Data-Collection and Data-Analysis Procedures

Data collection began between March and October 2022, after approval by the Research Ethics Committee, CAAE: 55034221.5.0000.5411, Report: 5.528.098.

The data were obtained from a primary source, the Department of Academic Activities Management (DGAA), through a survey of the MV system of the Medical Informatics Center (CIMED) of 238 invoiced accounts of beneficiaries from seven HICs treated by the hospital’s outpatient hemodynamics service between January 1, 2019 and December 31, 2021. These accounts were printed so that all of them could be subjected to a retrospective financial audit by one of the researchers, a nurse with a degree in health care auditing. This intervention was based on multidisciplinary records available in the patient’s electronic medical chart in the MV system and in accordance with the contractual agreements in place.

In order to obtain standardized account analyses and thus reduce bias, a cost measurement tool for the procedures selected for this study was developed in collaboration with the nurse in charge of the hemodynamics service. It should be noted that the tool for measuring costs related to hospital consumables and medications was pre-tested, without the need to adapt it to a pilot account with diagnostic and therapeutic procedures performed by the service. The OPSMs were based on the surgical description recorded in the electronic medical record, using for comparison the prices in force at the time and records from the Brazilian Health Regulatory Agency (Anvisa) to identify the product classification.

Six BGs comprised the audited hospital bill: room fees, equipment fees, medication fees, material fees, orthoses, prostheses, and special materials (OPSMs), and gas therapy.

After the analysis unit was audited, the data were entered into an Excel spreadsheet in columns for billed and audited items for comparative analysis.

This study used chargemaster values for materials, medications, OPSMs, and room and equipment charges associated with the procedures performed in the hemodynamics service.

The items listed in the BGs related to the care and/or procedures provided in the retrospective data collection were identified based on a detailed analysis of the medical and nursing notes in the beneficiary’s electronic medical record.

### Statistical Analysis

Descriptive statistics were used to analyze variables related to the characterization of beneficiaries within the set of billing accounts.

To analyze billing discrepancies by billing group, diagnostic and therapeutic procedures were compared with respect to the distribution of items in the “billed = audited”, “billed < audited”, and “billed > audited” ranges in the BGs using nonparametric Chi-square or Fisher’s exact tests. In addition, these procedures were compared for differences between billed and audited values using the nonparametric Mann-Whitney test for independent samples.

Statistically significant differences were considered when p < 0.05. The analysis was performed using the SPSS software, version 21.

The General Price Index - Market of Getúlio Vargas Foundation (IGP-M-FGV) was used to adjust the values in the final balance sheet, considering the initial period as July 2020 and the final period as September 2024^(19)^.

## RESULTS

### Characterization of Beneficiaries in the Unit of Analysis

Most beneficiaries of HICs treated in the outpatient hemodynamics service from January 1, 2019, to December 31, 2021, and included in the unit of analysis in this study, were males (60.5%) and aged ≥ 60 years (64.7%). Diagnostic confirmatory procedures (66.0%) predominated among medical requests, followed by therapeutic procedures (34.0%), mainly focused on diseases of the circulatory system (96.2%), with ischemic heart disease being the most common (86.1%). Cardiac catheterization was the most common procedure (61.8%), followed by cardiac angioplasty with cardiac stenting (29.0%), predominantly performed via radial access (84.9%) (Tables 1, 2 and 3).

**Table 1:** Beneficiaries treated from January 1, 2019, to December 31, 2021, by private health insurance in the outpatient hemodynamics service of a public hospital in the state of São Paulo, according to gender, age in years, type of procedure, and access route - Botucatu, SP, Brazil, 2024.

Gender (N = 238)	N	%
Female	94	39.5
Male	144	60.5
**Age (in years)** (N = 238)		
20 to 29	2	0.8
30 to 39	5	2.1
40 to 49	18	7.6
50 to 59	59	24.8
≥60	154	64.7
**Types of procedures** (N = 238)		
Cardiac catheterization	147	61.8
Cardiac angioplasty with a stent	69	29.0
Angiography	10	4.2
Chemoembolization	5	2.1
Surgical interatrial correction	3	1.3
Cardiac angioplasty with a stent + cerebral angioplasty with a stent	2	0.8
Transcatheter aortic valve prosthesis implant	2	0.8
**Access routes for exams** (N = 238)		
Radial	202	84.9
Femoral	33	13.9
Brachial	2	0.8
No record	1	0.4

**Table 2:** Diagnoses by groups and chapters of the International Classification of Diseases (ICD-10)*, recorded in test requests for beneficiaries of private health insurance, treated from January 1, 2019 to December 31, 2021, in the outpatient hemodynamics service of a public hospital in the state of São Paulo - Botucatu, SP, Brazil, 2024. (N = 238).

Groups	Codes (ICD)*	N	%
Chapter IX. Circulatory System Diseases*		229	96.2
- Ischemic heart diseases	I20, I21, I200, I209, I210, I211, I214, I219, I240, I251, I255	205	86.1
- Other forms of heart diseases	I352, I500	14	5.9
- Cerebrovascular diseases	I671, I620, I61	8	3.4
- Diseases of the arteries, arterioles, and capillaries	I702	2	0.8
Chapter II. Neoplasms [tumors]*		5	2.1
- Malignant neoplasms of digestive organs	C24, C220	5	2.1
Chapter XVII. Congenital malformations, deformities, and chromosomal anomalies*		3	1.3
- Congenital malformations of the circulatory system	Q211	3	1.3
Chapter VI. Nervous System Diseases*		1	0.4
- Episodic and paroxysmal disorders	G459	1	0.4
**Total**		**238**	**100.0**

*OMS, OPAS.

CID-10 - Classificação Estatística Internacional de Doenças e Problemas Relacionados à Saúde. 10ª ed. OMS, OPAS, editores. São Paulo: Edusp; 2017. 1200 p.

**Table 3:** Distribution of procedures by diagnostic and therapeutic groups of tests performed on beneficiaries of private health insurance, treated from January 1, 2019 to December 31, 2021, in the hemodynamics outpatient service of a public hospital in the state of São Paulo - Botucatu, SP, Brazil, 2024. (N = 238).

Procedures	Diagnostic		Therapeutic		Total	
	N	%	N	%	N	%
Cardiac catheterization	147	61.8	–	–	147	61.8
Cardiac angioplasty with stent placement	–	–	69	29.0	69	29.0
Angiography	10	4.2	–	–	10	4.2
Chemoembolization	–	–	5	2.1	5	2.1
Surgical interatrial correction	–	–	3	1.3	3	1.3
Cerebral angioplasty with stent placement	–	–	2	0.8	2	0.8
Transcatheter aortic valve prosthesis placement	–	–	2	0.8	2	0.8
**Total**	**157**	**66.0**	**81**	**34.0**	**238**	**100.0**

### Discrepancies in Invoices by Billing Group

In the room, medication, materials, OPSMs and gas therapy groups, there was a statistically significant difference between diagnostic and therapeutic procedures in relation to the distribution of items between the ranges: “billed = audited”, “billed < audited”, and “billed > audited”. In the room, medication, OPSMs, and gas therapy groups, the “billed < audited” range was more frequent among therapeutic procedures, while the same range was less frequent among therapeutic procedures in the materials group (Table 4).

**Table 4:** Comparisons between accounts billed by “non-nurses” and audited by nurses, according to billing groups - BGs (room, equipment, medications, materials, OPSMs, and gas therapy), for beneficiaries of private health insurance, treated from January 1, 2019, to December 31, 2021, in the outpatient hemodynamics service of a public hospital in the state of São Paulo - Botucatu, SP, Brazil, 2024.

Comparisons	General^(*)^		Diagnostic		Therapeutic		p-value^(**)^
	n	%	n	%	n	%	
**Billing Group (BG) Room Fees per unit (n = 238)**							
Billed = Audited	184	77.3	143	91.1	41	50.6	< 0.001
Billed < Audited	42	17.6	8	5.1	34	42.0	
Billed > Audited	12	5.0	6	3.8	6	7.4	
**Subtotal**	238	100.0	157	100.0	81	100.0	
**BG equipment fee per unit (n = 238)**							
Billed = Audited	43	53.8	30	54.5	13	52.0	0.870
Billed < Audited	36	45.0	24	43.6	12	48.0	
Billed > Audited	1	1.2	1	1.8	0	0.0	
**Subtotal**	80	100.0	55	100.0	25	100.0	
**BG medication fee per unit (n = 238)**							
Billed = Audited	316	18.1	180	25.7	136	13.0	< 0.001
Billed < Audited	901	51.7	343	48.9	558	53.5	
Billed > Audited	527	30.2	178	25.4	349	33.5	
**Subtotal**	1.744	100.0	701	100.0	1.043	100.0	
**BG materials fee per unit (n = 238)**							
Billed = Audited	1.293	21.3	696	17.8	597	27.6	< 0.001
Billed < Audited	3.695	60.9	2.460	62.9	1.235	57.1	
Billed > Audited	1.083	17.8	753	19.3	330	15.3	
**Subtotal**	6.071	100.0	3.909	100.0	2.162	100.0	
**BG orthoses, prostheses and special materials (OPSMs) per unit (n = 238)**							
Billed = Audited	101	6.5	33	4.4	68	8.5	< 0.001
Billed < Audited	734	47.3	334	44.3	400	50.0	
Billed > Audited	718	46.2	386	51.3	332	41.5	
**Subtotal**	1.553	100.0	753	100.0	800	100.0	
**BG gas therapy per unit (n = 238)**							
Billed = Audited	25	55.6	24	88.9	1	5.6	< 0.001
Billed < Audited	18	40.0	3	11.1	15	83.3	
Billed > Audited	2	4.4	0	0.0	2	11.1	
**Subtotal**	45	100.0	27	100.0	18	100.0	

^(*)^General = sum of units billed, used in diagnostic and therapeutic procedures;

^(**)^Chi-square test or Fisherss exact test.

Table 5 shows underbilling in the “billed < audited” range in 100% of BGs, regardless of diagnostic or therapeutic procedures, with a cumulative total of R$ 1,024,692.00, which, adjusted to the values until September 2024, would correspond to R$ 1,344,125.00. Among the cases of undue charges, “billed > audited”, there is a cumulative total of R$ 647,828.00.

**Table 5:** Balance in Brazilian currency (Real - R$), adjusted by the IGP-M (FGV) index, of accounts billed by “non-nurses”, and audited by nurses, by billing group ranges (BGs), referring to diagnostic and therapeutic procedures performed on beneficiaries of private health insurance, from January 1, 2019 to December 31, 2021, in outpatient hemodynamics services at a public hospital in the State of São Paulo - Botucatu, SP, Brazil, 2024 (N = 238).

Billing Groups (BG) by Ranges	General in R$*				Diagnostic Procedures in R$				Therapeutic Procedures in R$				p-value**
	md (1Q; 3Q)^†^	TotBill^‡^	TotAud^II^	Dif^¶^	md (1Q ; 3Q)^†^	TotBill^‡^	TotAud^II^	Dif^¶^	md (1Q ; 3Q)^†^	TotBill^‡^	TotAud^‡^	Dif^¶^	
**Billing Group (BG) room fee per unit**													
Billed < Audited	-153 (-300; -144)	22,253.00	31,615.00	-9,362.00	-40 (-142; -33)	3,170.00	3,775.00	-605.00	-153 (-309; -153)	19,352.00	27,840.00	-8,488.00	*< 0.001*
Billed > Audited	226 (138; 687)	11,196.00	6,921.00	4,275.00	144 (123; 611)	4,532.00	2,718.00	1,814.00	358 (146; 726)	6,664.00	4,203.00	2,461.00	*0.394*
**BG equipment fee per unit**													
Billed < Audited	- 28 (-28; -28)	0.00	1,091.00	-1,091.00	- 28 (-28; -28)	0.00	755.00	-755.00	-28 (-28; -28)	0.00	336.00	-336.00	*1.000*
Billed > Audited	28 (28; 28)	28.00	0.00	28.00	28 (28; 28)	28.00	0.00	28.00					
**BG medication fee per unit**													
Billed < Audited	-11 (-189; -5)	24,442.00	159,147.00	-134,705.00	-11 (-162; -5)	12,118.00	79,242.00	-67,124.00	-12 (-319; -5)	12,323.00	79,904.00	-67,581.00	*0.320*
Billed > Audited	12 (3; 210)	75,975.00	4,480.00	71,495.00	12 (3; 109)	48,390.00	3,268.00	45,122.00	12 (5; 332)	27,585.00	1,211.00	26,374.00	*0.062*
**BG materials fee per unit**													
Billed < Audited	-21 (-185; -7)	21,000.00	335,711.00	-314,711.00	-21 (-185; -7)	14,668.67	212,657.00	-197,988.00	-21 (-196; -8)	6,331.22	123,054.00	-116,722.00	*0.120*
Billed > Audited	15 (5; 33)	46,433.00	1,871.00	44,562.00	17 (5; 37)	34,794.40	915.00	33,879.00	13 (5; 26)	11,638.77	956.00	10,682.00	*0.023*
**BG orthoses, prostheses and special materials (OPSMs) fee per unit**													
Billed < Audited	-172 (-258; -92)	84,649.00	648,804.00	-564,154.00	-142 (-178; -63)	17,938.00	63,800.00	-45,862.00	-247 (-661; -154)	66,710.00	585,002.00	-518,292.00	*< 0.001*
Billed > Audited	60 (20; 214)	589,097.00	61,672.00	527,425.00	44 (19; 71)	28,844.00	2,445.00	26.398.00	213 (44; 1024)	560,253.00	59,225.00	501,027.00	*< 0.001*
**BG gas therapy fee per unit**													
Billed < Audited	-27 (-54; -14)	0.00	668.41	-668,41.00	-27 (-54; xxx)	0.00	109.28	-109.28	-27 (-54; -14)	0.00	559.13	-559.00	*1.000*
Billed > Audited	20 (14; xxx)	41.90	0.00	41.90				0	20 (14; xxx)	41.90	0.00	41.00	*0.634*
**Balance in Brazilian currency (Real – R$) for the period of billed and audited accounts. adjusted by the IGP-M (FGV) index, as updated^††^**													
Billed < Audited				-1,024,692.00				-312,444.00				-711,980.00	
Billed < Audited corrected^††^				-1,344,125.00				-454,895.00				1,036,590.00	
Billed > Audited				647,828.00				107,242.00				540,587.00	
Billed < Audited corrected^††^				943,189.00				156,136.00				787,054.00	

*General = sum of units billed, used in diagnostic and therapeutic procedures;

^†^md = median (1Q = First quartile and 3Q = Third quartile);

^‡^TotBill = Total billed;

^II^TotAud = Total audited;

^¶^Dif = Difference between billed and audited values in Brazilian currency (R$)

**Mann-Whitney test for independent samples;

^††^Values adjusted by the IGP-M index (FGV), considering the initial period in 07/2020 and the final period in 09/2024^(19)^.

The difference between the Q1 and Q3 medians is called the interquartile range (IQR)^†^. The larger the IQs between the groups being compared, the greater the variability of the data and, consequently, the lower the chances of detecting statistically significant differences.

Considering the “billed < audited” range, therapeutic procedures showed significantly greater differences compared to diagnostic procedures in the room and OPSMs groups. In the “billed > audited” range, therapeutic procedures showed significantly smaller differences in the materials group and larger differences in the OPSMs group (Table 5).

## DISCUSSION

In this study, the epidemiologic profile of HIC beneficiaries treated at the hemodynamics outpatient service confirmed that described in other studies conducted in Brazil. In such studies, male^(20)^, elderly (60 years or older) patients with circulatory disease, mainly ischemic heart disease, requiring cardiac catheterization and angioplasty with stents^(20,21)^ and the femoral artery as the main route of catheter access^(20)^, were predominant.

In addition, these patients had a significant statistical association with coronary artery disease (CAD), overweight or obesity, previous metabolic comorbidities, and use of medications to control hypertension and diabetes mellitus^(21)^. These are chronic noncommunicable diseases (NCDs) that could be better controlled in primary care settings^(20)^.

Brazil is one of the countries with the largest elderly population^(22)^, which will triple by 2050, and it is undergoing a demographic change accompanied by an epidemiological transition characterized by the multimorbidity of NCDs^(23)^.

This is one of the reasons why Brazil has one of the highest prevalence of multimorbidity in the world, at approximately 70%, with the elderly most affected, similarly to what occurs in high-income countries, such as Spain and Finland. However, the Brazilian context of risk can be considered, where the epidemiological transition has been rapid and with many challenges to overcome^(24)^, related to social inequalities and increasing health care costs^(25)^.

On the other hand, the results of this study corroborated the hypothesis that the lack of prior nursing audits may contribute to greater financial and economic losses for institutions, especially due to systematic underbilling of all BGs, resulting in lower financial revenues from hemodynamics services.

This study indicated that discrepancies in room fees were related to inappropriate charges for room size according to the anesthesia level of the procedure performed and approved by the HIC, showing a lack of knowledge by the administrative staff involved in the hospital billing process.

The underbilling in the gas therapy group resulted from a failure to review multidisciplinary treatment records, as was the case in the equipment group, demonstrating a lack of knowledge or review by “non-nurse” staff.

These discrepancies were exacerbated in therapeutic procedures related to materials and medications, which are considered to have a higher financial return and are, therefore, more profitable, since they include items in greater quantities and are billed according to the rates practiced in the supplemental health care market.

The underbilling of total consumables was due to the lack of recording in the beneficiary’s medical chart or billing account, as well as the lack of measures to control expenditures.

With regard to medications, discrepancies occurred for three main reasons: quantity, incorrect entry in the system, and lack of record in the medical chart, which were the main reasons for underbilling. It should be noted that the entry of items in the system follows a logic based on the presentation of the medication, with some being entered in ampoules and others in milligrams or milliliters.

Since all medical and nursing care generates costs for the hospital, it is important that all care provided be clearly and accurately described so that there are no financial losses due to disallowances, since payments are made on the basis of supporting documentation, i.e., descriptions, checks, and reports proving that the procedures were performed^(26)^.

The audit identified two types of billing discrepancies in the OPSM billing group. The first was related to the misclassification of materials according to the Simpro table^(16)^, since hospital consumables were billed as OPSMs. The second discrepancy indicated that OPSM billing was based on the institution’s purchase price (bid), specifically in accounts related to the insurance company with the largest number of beneficiaries, without respecting the contractual clause between the hospital and the HICs for billing based on quotes from registered suppliers. Since the bid prices were significantly lower than the quoted value, situations arose that contributed to the loss of financial revenue.

These occurrences in the OPSM authorization and billing process demonstrated contractual and conceptual misinformation among “non-nurse” staff, as they are professionals without health care training and without specific training to perform their duties.

The presence of a technical professional with health care and administrative knowledge, such as a nurse auditor, can help minimize these gaps. In addition, the presence of such a professional in the process of purchasing and evaluating these OPSMs has ensured the quality of the care provided and the cost-benefit ratio through systematic analysis of medical records and concurrent auditing. These procedures make it possible to detect inconsistencies that, when resolved, increase patient safety and reduce accounting and financial losses for the organization^(27)^.

Recent studies have shown that high rates of incomplete information entered into patient records by nurses have serious consequences for care delivery, administration, and finances, which could be minimized by raising awareness among health care professionals through nursing audits^(28,29)^.

A reflective study on nursing auditing as a tool to improve care also emphasized that the procedure provides management with information for decision making, ensures financial balance with best clinical practices and is a tool that allows the implementation of actions to improve care provision processes through educational activities for the team^(30)^.

Concerns about health care cost management are not unique to HICs. In the SUS, the National Health Cost Management Program (PNGC), created by the Ministry of Health, is an important management tool for the public health system^(31)^.

One of the objectives of PNGC and the Cost Calculation and Management System of the Brazilian Unified Health System (ApuraSUS) is to compare the estimated costs of the services provided and their respective revenues, as well as the waste generated by inefficient use to produce the product. The calculation and control of health care costs allow the implementation of corrective measures to improve the performance of units, based on the redefinition of priorities, increased productivity and rationalization of the use of resources, among other administrative measures^(31)^.

Thus, the interfaces between SUS and health insurance companies are similar in terms of financial and economic viability in the provision of care, as they require greater efficiency and less waste in the allocation of material and financial resources.

Aligning the audit with the institution’s policies and strategic planning strengthens the implementation of improvements and the development of new resources capable of inducing continuous training of clinical and health care personnel for better management of existing resources, as well as comparative studies of the best forms of remuneration.

However, this study contributes to the construction of the role of the financial audit nurse, based on technical, scientific and managerial knowledge to perform in care-provision and administrative processes in the health care market. This is an executive professional, assigned to the senior management of a health care institution, with the ability to support decision making, not only in the retrospective auditing of accounts, but throughout the entire process of the Supplemental Health Care Center, such as: accreditation of new providers; approval of procedures; negotiation of contracts, pricing tables and procedure packages.

Auditors are necessary to control costs and assist managers in analyzing hospital bills, focusing on the value of health care, not just on reducing expenses. Cost control by auditors is aimed at achieving comprehensive health care for beneficiaries, with the goal of maximizing available human and material resources^(8)^.

For nursing as a profession, the emerging role of the nurse financial auditor contributes to the advancement of performance opportunities with autonomous participation and implementation of decisions for quality care at the senior management level of health care institutions, a position rarely held by nurses. It is recommended that undergraduate nursing programs, as well as graduate programs in nursing management, include training in economic and financial literacy in health care so that nurses can work in public and private health services, as there are no processes without cost control.

These competencies can reduce the marginalization of nurses in processes where their care-provision work is subordinated to the decisions of other professionals, especially when the health sector is under pressure and nurses can contribute proactively, from those in a clinical role to those with managerial positions^(8)^.

Nursing education for the 21^st^ century requires a paradigm shift so that financial literacy becomes part of nurses’ professional skills, enabling them to contribute to addressing the economic and financial burden generated by population aging in health care systems due to the mandatory scope of diagnostic and therapeutic procedures.

The following are considered limitations to this study in terms of: (a) the lack of direct consideration of human resources, as they are indirectly included in the room charge; (b) data collection, due to the lack of authorization records for the period 2019; (c) methodological limitations of the retrospective design, which lacked a control group and possible biases in the use of secondary data; (d) the non-adoption of formal economic modeling, as it used descriptive statistics and non-parametric tests.

## CONCLUSION

The results of the study confirmed the hypothesis, finding that hospital bills submitted to retrospective audit by nurses specializing in this area increased hospital revenue, with underbilling detected in all BGs when performed by “non-nurses”. Finally, this study helps to demonstrate the need for training nurses with economic and financial literacy in health care, highlighting the importance of the emerging role of the nurse financial auditor in the health care marketplace and consequently contributing to better quality care. In addition, more robust comparative studies are recommended to assess the impact of nurse financial auditing in different hospital settings.

## DATA AVAILABILITY

The database from this research will be available upon request to the primary researcher, who can be contacted via e-mail at silvia.meneguin@unesp.br.
