# Supplementary material for: Nurse performance in hospital financial auditing: a single case study
Source: Rev Esc Enferm USP. 2025 Jul 7;59:e20240391. doi: 10.1590/1980-220X-REEUSP-2024-0391en (PMC12258053; doi:10.1590/1980-220X-REEUSP-2024-0391en)
Supplement: Translation

# Translation: Desempenho do enfermeiro na auditoria financeira hospitalar: estudo de caso único

**DOI:** 10.1590/1980-220X-REEUSP-2024-0391pt

**Authors:** Silvia Helena Meneguin, Vânia dos Santos Nunes Nogueira, Silvia Cristina Mangini Bocchi

**Affiliations:** 1 Universidade Estadual Paulista, Faculdade de Medicina de Botucatu, Botucatu, SP, Brasil.; 2 Universidade Estadual Paulista, Faculdade de Medicina de Botucatu, Departamento de Clínica Médica, Botucatu, SP, Brasil.; 3 Universidade Estadual Paulista, Faculdade de Medicina de Botucatu, Departamento de Enfermagem, Botucatu, SP, Brasil.

**Keywords:** Papel do Profissional de Enfermagem, Auditoria de Enfermagem, Saúde Suplementar, Administração Financeira, Economia e Organizações de Saúde

## Abstract

**Objetivo:**

Analisar o desempenho do enfermeiro na auditoria financeira retrospectiva, comparada à realizada por “não enfermeiros”, para a remuneração de um hospital por operadoras de planos da saúde suplementar.

**Método:**

Estudo de caso único, quantitativo, retrospectivo, documental e analítico, tendo como unidade de análise o conjunto de 238 contas contábeis faturadas por “não enfermeiros” e auditadas por enfermeiro, de procedimentos ambulatoriais no serviço de hemodinâmica, em um hospital público do estado de São Paulo, entre 2019 e 2021.

**Resultados:**

Constatou-se subfaturamento na faixa “faturado < auditado”, em 100% dos grupos de faturamento, independentemente dos procedimentos realizados, com um acumulado de R$ 1.024.692,00, que atualizado até setembro de 2024, corresponderia a R$ 1.344.125,00. Nos casos de cobrança indevida, “faturado > auditado”, houve um acumulado de R$ 647.828,00.

**Conclusão:**

A auditoria financeira retrospectiva, realizada por enfermeiro, aumenta a receita hospitalar. Por fim, este estudo contribui ao demonstrar a necessidade de formação desse profissional com competência econômico-financeira em saúde, vislumbrando a importância do papel emergente do enfermeiro auditor financeiro no mercado da saúde.

## INTRODUÇÃO

O mercado da saúde, ao deparar-se com o risco agravado de insustentabilidade financeira e econômica de seus negócios, devido à celeridade no aumento do custo assistencial^(1)^, vem se mobilizando pelo gerenciamento de orçamentos reduzidos para o equilíbrio entre custos, despesas e receitas, visando assegurar a sobrevivência de suas organizações.

A escassez de recursos financeiros decorre dos avanços tecnológicos e da mudança demográfica, acompanhada por transição epidemiológica para população idosa^(2)^. Desse contexto emerge a necessidade de encontrar gestores da área da saúde capacitados para prover um sistema eficaz de gerenciamento de custo, sem prejuízo para a qualidade assistencial, tendo como fim a eficiência na distribuição dos recursos^(3)^.

Sabe-se que os cuidados de enfermagem consomem aproximadamente um terço do orçamento hospitalar^(4)^, requerendo enfermeiros com conhecimentos e habilidades na tomada de decisões acerca da estrutura, processos e resultados institucionais. A propósito, para o gerenciamento de serviços de enfermagem, torna-se necessária a formação de enfermeiros instrumentalizados sobre custos, como mais uma ferramenta a ser utilizada nos processos decisórios^(3)^.

Nesse sentido, a literatura tem corroborado a necessidade do alcance da competência econômico-financeira por enfermeiros que vislumbram ou atuam na função de gestores financeiros em instituições de saúde^(5-7)^, para que desenvolvam ações de sustentabilidade da assistência oferecidas por essas organizações^(8)^ e demonstrando ser esta uma área de atuação gerencial promissora para o profissional, na função de auditor^(9)^.

Nesta pesquisa empregou-se a auditoria retrospectiva, operacionalizada após a alta hospitalar e executada mediante a conferência sistemática da cobrança pelos serviços hospitalares com base no prontuário do paciente^(10)^, para avaliar distorções de caráter administrativo e assistencial de um serviço de hemodinâmica.

São escassos artigos científicos que vinculam a economia da saúde com a implementação de auditorias financeiras internas por enfermeiros^(8,11,12)^.

Em face ao exposto, emergiu a pergunta desta pesquisa: Como a atuação do enfermeiro na auditoria financeira retrospectiva de contas hospitalares, faturadas por colaboradores “não enfermeiros”, pode contribuir para a receita hospitalar?

Presumiu-se, como hipótese, que adotar o processo de auditagem prévia em contas hospitalares por enfermeiros aumentaria a efetividade na gestão do faturamento.

Para testar essa hipótese, delineou-se como objetivo: analisar o desempenho do enfermeiro na auditoria financeira retrospectiva, comparada à realizada por “não enfermeiros”, na remuneração de um hospital por operadoras de planos da saúde (OPSs).

## MÉTODO

### Desenho de Estudo

Trata-se de estudo de caso único^(13)^, quantitativo, retrospectivo, documental e analítico. A unidade de análise envolveu a avaliação econômica em saúde e, portanto, empregou-se o roteiro CHEERS para relatá-lo^(14)^.

### Local e Unidade de Análise

A unidade de análise constituiu-se do conjunto de 238 contas faturadas, no modelo de pagamento *Fee for Service* (conta aberta dos itens de cobrança)^(15)^, de 01/01/2019 a 31/12/2021, por “não-enfermeiros” e auditadas por enfermeiro. Essas contas decorreram de beneficiários de sete OPSs, submetidos a procedimentos diagnósticos e terapêuticos, no serviço de hemodinâmica ambulatorial de um hospital público do estado de São Paulo, Brasil.

A seleção desse conjunto derivou de 631 contas contábeis faturadas, excluindo-se 393 que tiveram seus beneficiários internados ou provenientes de outros modelos de pagamento. A contento, a análise adotada deu-se na perspectiva hospitalar e sob a ótica do impacto financeiro auditado pelo enfermeiro.

Ressalta-se que a escolha do local e do período para este estudo se deu em razão de os procedimentos diagnósticos e terapêuticos serem de alto custo, associados à ausência de controle anterior à implantação de auditoria retrospectiva por enfermeiro nas contas contábeis. A contento, de 01/01/2019 a 31/12/2021, essas contas eram faturadas por profissionais administrativos, sem qualquer formação na área da saúde e, portanto, denominados, neste estudo, de colaboradores “não enfermeiros”.

O serviço de hemodinâmica se encontra inserido no Núcleo de Procedimentos Diagnósticos e Terapêuticos do hospital, com média de 3.700 exames mensais, majoritariamente de beneficiários do Sistema Único de Saúde (SUS), e apenas 6% deles são de conveniados de OPSs. O lucro desse serviço é destinado às melhorias da qualidade da assistência prestada pela instituição.

### Processo de Faturamento e Auditoria da Conta Contábil

A conta contábil dos serviços prestados pelo setor de hemodinâmica discrimina-se por grupos de faturamento (GFs), dentre eles: taxa de sala; gasoterapia; equipamentos; materiais; medicamentos; órtese, prótese e materiais especiais (OPMEs), assim como honorários médicos.

A precificação de diárias e taxas seguem os contratos de prestação de serviço pactuados entre as operadoras e o hospital, assim como as tabelas de referência no mercado de saúde, Simpro^(16)^ e Brasíndice^(17)^, para remuneração de materiais e medicamentos. Para os OPMEs, contudo, empregam-se os valores de cotação do fornecedor.

O faturamento da conta se inicia com a admissão do beneficiário no serviço, solicitando-se autorização às OPSs, de acordo com as necessidades pré- e pós-procedimentos, e só finaliza com o envio da fatura de pagamento às operadoras, após submetida à auditoria por enfermeiro especializado nessa área.

Essa auditoria visa revisar a conta, de acordo com o contrato de prestação de serviço acordado com a OPS, os registros assistenciais multidisciplinares no prontuário eletrônico do beneficiário e as autorizações pertinentes a todo o processo de atendimento. Tal procedimento permite ajustes conforme a necessidade de inclusão ou exclusão de itens na conta, para que seja enviada à OPS com o rigor e a lisura de uma prestação de serviço de qualidade.

O processo pós-faturamento envolve vários setores, por estar atrelado ao pagamento e ao repasse de honorários médicos, que acompanham o desfecho do recurso de glosas para se dar baixa no faturamento do hospital.

### Variáveis

*De caracterização dos beneficiários da unidade de análise:* sexo (F/M); idade por faixas etárias em anos (20 a 29, 30 a 39, 40 a 49, 50 a 59 e ≥ 60); diagnósticos médicos em solicitações de exames (classificados por grupos e capítulos do Código Internacional de Doenças - CID 10)^(18)^; classificação de procedimentos diagnósticos e terapêuticos (angiografia, cateterismo cardíaco, angioplastia com *stent* cardíaco e cerebral, correção cirúrgica interatrial, quimioembolização e implante transcateter de prótese valvar); via de acesso dos exames (braquial, radial e femoral).

*Independentes:* os tipos de procedimentos, agrupados em terapêuticos e diagnósticos, realizados no serviço de hemodinâmica.

*Dependentes:* resultados entre faturados por colaborador administrativo “não enfermeiro” (e não da área da saúde) e auditados por enfermeiro com formação em auditoria, de itens classificados nas faixas de distribuição “faturado = auditado”, “faturado < auditado” e “faturado > auditado”, para esta pesquisa, nos GFs, denominados taxas: de sala, de equipamentos, de medicamentos, de materiais, de OPMEs e de gasoterapia. Considerou-se eficiência do enfermeiro auditor, quando este detectou itens na faixa “faturado < auditado”, uma vez que os itens agrupados na faixa “faturado > auditado”, em sua maioria, são glosados.

### Procedimentos Éticos, de Coleta e Análise dos Dados

A coleta de dados se iniciou entre março e outubro de 2022, após aprovação do projeto de pesquisa por Comitê de Ética em Pesquisa, CAAE: 55034221.5.0000.5411, parecer: 5.528.098.

Os dados decorreram de fonte primária do Departamento de Gestão de Atividades Acadêmicas (DGAA), por levantamento no Sistema MV do Centro de Informática Médica (CIMED), de 238 contas faturadas de beneficiários de sete OPSs, atendidos de 01/01/2019 a 31/12/2021, pelo serviço de hemodinâmica ambulatorial do hospital. Imprimiram-se essas contas para que todas fossem submetidas à auditoria financeira retrospectiva por uma das pesquisadoras, enfermeira com pós-graduação na área de Auditoria em Saúde. Essa intervenção deu-se a partir de registros multidisciplinares disponíveis no prontuário eletrônico do paciente no Sistema MV e conforme os acordos contratuais vigentes.

Para a obtenção de análises das contas de forma padronizada e, portanto, com redução de vieses, conjuntamente com o enfermeiro responsável pelo serviço de hemodinâmica, elaborou-se instrumento de mensuração de gastos para os procedimentos eleitos para esta pesquisa. Ressalta-se que o instrumento para mensuração de gastos, relativos aos itens materiais de consumo hospitalar e medicamentos passou por pré-teste, sem precisar readequá-lo a uma conta piloto com procedimentos diagnósticos e terapêuticos realizados pelo serviço. Os OPMEs deram-se de acordo com o registro da descrição cirúrgica no prontuário eletrônico, sendo utilizados para comparação os valores de cotação da época e registros da Agência Nacional de Vigilância Sanitária (Anvisa) para identificação da classificação do produto.

Seis GFs compuseram a conta hospitalar auditada: taxa de sala; taxa de equipamentos; taxa de medicamentos; taxa de materiais; órtese, prótese e materiais especiais (OPMEs); gasoterapia.

Após a auditoria da unidade de análise, os dados foram planilhados no Excel, em colunas de faturados e auditados para análise comparativa.

Esta pesquisa utilizou-se de valores de remuneração dos recursos materiais, de medicamentos, de OPMEs e de taxas de sala e equipamentos inerentes aos procedimentos executados no serviço de hemodinâmica.

Identificaram-se os itens discriminados dos GFs, relacionados aos atendimentos e/ou procedimentos da coleta de dados retrospectiva, a partir da análise detalhada dos registros médicos e de enfermagem no prontuário eletrônico do beneficiário.

### Análise Estatística

Empregou-se a estatística descritiva para análise das variáveis relativas à caracterização dos beneficiários do conjunto das contas contábeis.

Para analisar as discordâncias nas faturas por grupo de faturamento, compararam-se os procedimentos diagnósticos e terapêuticos em relação à distribuição dos itens nas faixas “faturado = auditado”, “faturado < auditado” e “faturado > auditado” nos GFs, por meio dos testes não paramétricos Qui-quadrado ou Exato de Fisher. Ademais, compararam-se esses procedimentos em relação às diferenças entre valores faturados e auditados, por teste não paramétrico de Mann-Whitney para amostras independentes.

Consideraram-se diferenças estatisticamente significativas se p < 0,05. Análise realizada com o software SPSS, versão 21*.*

Empregou-se o Índice Geral de Preços – Mercado, da Fundação Getúlio Vargas (IGP-M-FGV), para a correção dos valores no balanço final, considerando o período inicial o mês de julho de 2020 e o período final o mês de setembro de 2024^(19)^.

## RESULTADOS

### Caracterização dos Beneficiários da Unidade de Análise

A maioria dos beneficiários de OPSs, atendidos de 01/01/2019 a 31/12/2021 no serviço ambulatorial de hemodinâmica e contidos na unidade de análise deste estudo, é do sexo masculino (60,5%) e está na faixa etária ≥ 60 anos (64,7%). Nas solicitações médicas prevalecem procedimentos de confirmação diagnóstica (66,0%), seguidos por terapêuticos (34,0%), voltados majoritariamente às doenças do aparelho circulatório (96,2%), sendo o grupo das doenças isquêmicas do coração o de maior incidência (86,1%). A contento, predomina o cateterismo cardíaco (61,8%), seguido da angioplastia com *stent* cardíaco (29,0%), realizados preponderantemente por acesso radial (84,9%) (Tabelas 1, 2 e 3).

**Tabela 1:** Beneficiários atendidos, de 01/01/2019 a 31/12/2021, por operadoras de planos da saúde suplementar, no serviço de hemodinâmica ambulatorial de hospital público do estado de São Paulo, segundo: sexo, idade em anos, tipos de procedimentos e vias de acesso - Botucatu, SP, Brasil, 2024.

Sexo (N = 238)	N	%
Feminino	94	39,5
Masculino	144	60,5
**Idade (em anos)** (N = 238)		
20 a 29	2	0,8
30 a 39	5	2,1
40 a 49	18	7,6
50 a 59	59	24,8
≥60	154	64,7
**Tipos de procedimentos** (N = 238)		
Cateterismo cardíaco	147	61,8
Angioplastia com *stent* cardíaco	69	29,0
Angiografia	10	4,2
Quimioembolização	5	2,1
Correção cirúrgica interatrial	3	1,3
Angioplastia com *stent* cardíaco + angioplastia com *stent* cerebral	2	0,8
Implante transcateter de prótese valvular aórtica	2	0,8
**Via de acesso dos exames** (N = 238)		
Radial	202	84,9
Femoral	33	13,9
Braquial	2	0,8
Sem anotação	1	0,4

**Tabela 2:** Diagnósticos por grupos e capítulos, do Código Internacional de Doenças (CID 10)*, registrados nas solicitações de exames de beneficiários de operadoras de planos da saúde suplementar, atendidos de 01/01/2019 a 31/12/2021, no serviço de hemodinâmica ambulatorial de hospital público do estado de São Paulo - Botucatu, SP, Brasil, 2024 (N = 238).

Grupos	Códigos (CID)*	N	%
Capítulo IX. Doenças do Aparelho Circulatório*		229	96,2
- Doenças isquêmicas do coração	I20, I21, I200, I209, I210, I211, I214, I219, I240, I251, I255	205	86,1
- Outras formas de doença do coração	I352, I500	14	5,9
- Doenças cerebrovasculares	I671, I620, I61	8	3,4
- Doenças das artérias, das arteríolas e dos capilares	I702	2	0,8
Capítulo II. Neoplasias [tumores]*		5	2,1
- Neoplasias malignas dos órgãos digestivos	C24, C220	5	2,1
Capítulo XVII. Malformações congênitas, deformidades e anomalias cromossômicas*		3	1,3
-Malformações congênitas do aparelho circulatório	Q211	3	1,3
Capítulo VI. Doenças do Sistema Nervoso*		1	0,4
- Transtornos episódicos e paroxísticos	G459	1	0,4
**Total**		**238**	**100,0**

*OMS, OPAS.

CID-10 - Classificação Estatística Internacional de Doenças e Problemas Relacionados à Saúde. 10ª ed. OMS, OPAS, editores. São Paulo: Edusp; 2017. 1200 p.

**Tabela 3:** Distribuição dos procedimentos por grupos diagnósticos e terapêuticos de exames de beneficiários de operadoras de planos da saúde suplementar, atendidos de 01/01/2019 a 31/12/2021, no serviço de hemodinâmica ambulatorial, de hospital público do estado de São Paulo - Botucatu, SP, Brasil, 2024 (N = 238).

Procedimentos	Diagnósticos		Terapêuticos		Total	
	N	%	N	%	N	%
Cateterismo cardíaco	147	61,8	–	–	147	61,8
Angioplastia com *stent* cardíaco	–	–	69	29,0	69	29,0
Angiografia	10	4,2	–	–	10	4,2
Quimioembolização	–	–	5	2,1	5	2,1
Correção cirúrgica interatrial	–	–	3	1,3	3	1,3
Angioplastia com *stent* cerebral	–	–	2	0,8	2	0,8
Implante transcateter de prótese valvular aórtica	–	–	2	0,8	2	0,8
**Total**	**157**	**66,0**	**81**	**34,0**	**238**	**100,0**

### Discordâncias nas Faturaspor Grupo de Faturamento

Nos grupos sala, medicamentos, materiais, OPMEs e gasoterapia, houve diferença estatisticamente significativa entre os procedimentos diagnósticos e terapêuticos, em relação à distribuição dos itens entre as faixas: “faturado = auditado”, “faturado < auditado” e “faturado > auditado”. Nos grupos sala, medicamentos, OPMEs e gasoterapia, a faixa “faturado < auditado” é mais frequente entre os procedimentos terapêuticos, enquanto essa mesma faixa é menos frequente entre os procedimentos terapêuticos no grupo dos materiais (Tabela 4).

**Tabela 4:** Comparações entre as contas contábeis faturadas por “não enfermeiros” e auditadas por enfermeiro, conforme grupos de faturamento - GFs (sala, equipamentos, medicamentos, materiais, OPMEs e gasoterapia), de beneficiários de operadoras de planos da saúde suplementar, atendidos de 01/01/2019 a 31/12/2021, em serviço de hemodinâmica ambulatorial de hospital público do estado de São Paulo - Botucatu, SP, Brasil, 2024

Comparações	Geral(*)		Diagnóstico		Terapêutico		p-valor(**)
	n	%	n	%	n	%	
**Grupo de faturamento (GF) taxa de sala por unidade (n = 238)**							
Faturado = Auditado	184	77,3	143	91,1	41	50,6	< 0,001
Faturado < Auditado	42	17,6	8	5,1	34	42,0	
Faturado > Auditado	12	5,0	6	3,8	6	7,4	
**Subtotal**	238	100,0	157	100,0	81	100,0	
**GF taxa de equipamentos por unidade (n = 238)**							
Faturado = Auditado	43	53,8	30	54,5	13	52,0	0,870
Faturado < Auditado	36	45,0	24	43,6	12	48,0	
Faturado > Auditado	1	1,2	1	1,8	0	0,0	
**Subtotal**	80	100,0	55	100,0	25	100,0	
**GF taxa de medicamentos por unidade (n = 238)**							
Faturado = Auditado	316	18,1	180	25,7	136	13,0	< 0,001
Faturado < Auditado	901	51,7	343	48,9	558	53,5	
Faturado > Auditado	527	30,2	178	25,4	349	33,5	
**Subtotal**	1.744	100,0	701	100,0	1.043	100,0	
**GF taxa de materiais por unidade (n = 238)**							
Faturado = Auditado	1.293	21,3	696	17,8	597	27,6	< 0,001
Faturado < Auditado	3.695	60,9	2.460	62,9	1.235	57,1	
Faturado > Auditado	1.083	17,8	753	19,3	330	15,3	
**Subtotal**	6.071	100,0	3.909	100,0	2.162	100,0	
**GF órteses, próteses e materiais especiais (OPMEs) por unidade (n = 238)**							
Faturado = Auditado	101	6,5	33	4,4	68	8,5	< 0,001
Faturado < Auditado	734	47,3	334	44,3	400	50,0	
Faturado > Auditado	718	46,2	386	51,3	332	41,5	
**Subtotal**	1.553	100,0	753	100,0	800	100,0	
**GF gasoterapia por unidade (n = 238)**							
Faturado = Auditado	25	55,6	24	88,9	1	5,6	< 0,001
Faturado < Auditado	18	40,0	3	11,1	15	83,3	
Faturado > Auditado	2	4,4	0	0,0	2	11,1	
**Subtotal**	45	100,0	27	100,0	18	100,0	

^(*)^Geral = soma de unidades faturadas, utilizadas em procedimentos diagnósticos e terapêuticos;

(**)Teste de Qui-quadrado ou Exato de Fisher.

Consta na Tabela 5 subfaturamento na faixa “faturado < auditado” em 100% dos GFs, independentemente dos procedimentos diagnósticos ou terapêuticos, com um acumulado de R$ 1.024.692,00, que reajustado aos valores de até setembro de 2024 corresponderia a R$ 1.344.125,00. Já entre os casos de cobrança indevida, “faturado > auditado”, apresenta-se um acumulado de R$ 647.828,00.

**Tabela 5:** Balanço em moeda oficial brasileira (Real - R$), corrigido pelo índice IGP-M (FGV) das contas contábeis faturadas por “não enfermeiros” e auditadas por enfermeiro, por faixas de grupos de faturamento - GFs, referentes a procedimentos diagnósticos e terapêuticos, realizados em beneficiários de operadoras de planos da saúde suplementar, de 01/01/2019 a 31/12/2021, em serviço de hemodinâmica ambulatorial de hospital público do estado de São Paulo - Botucatu, SP, Brasil, 2024 (N = 238).

Grupos de faturamento (GF) por faixas	Geral em R$*				Procedimentos Diagnósticos em R$				Procedimentos Terapêuticos em R$				p-valor**
	md (1Q; 3Q)†	TotFat‡	TotAud^II^	Dif^¶^	md (1Q ; 3Q)†	TotFat‡	TotAud^II^	Dif^¶^	md (1Q ; 3Q)†	TotFat‡	TotAud‡	Dif^¶^	
**Grupo de faturamento (GF) de taxa de sala por unidade**													
Faturado < Auditado	–153 (–300; –144)	22.253,00	31.615,00	–9.362,00	–40 (–142; –33)	3.170,00	3.775,00	–605,00	–153 (–309; –153)	19.352,00	27.840,00	–8.488,00	*< 0,001*
Faturado > Auditado	226 (138; 687)	11.196,00	6.921,00	4.275,00	144 (123; 611)	4.532,00	2.718,00	1814,00	358 (146; 726)	6.664,00	4.203,00	2.461,00	*0,394*
**GF de taxa de equipamentos por unidade**													
Faturado < Auditado	– 28 (–28; –28)	0,00	1.091,00	–1.091,00	– 28 (–28; –28)	0,00	755,00	–755,00	–28 (–28; –28)	0,00	336,00	–336,00	*1,000*
Faturado > Auditado	28 (28; 28)	28,00	0,00	28,00	28 (28; 28)	28,00	0,00	28,00					
**GF de taxa de medicamentos por unidade**													
Faturado < Auditado	–11 (–189; –5)	24.442,00	159.147,00	–134.705,00	–11 (–162; –5)	12.118,00	79.242,00	–67.124,00	–12 (–319; –5)	12.323,00	79.904,00	–67.581,00	*0,320*
Faturado > Auditado	12 (3; 210)	75.975,00	4.480,00	71.495,00	12 (3; 109)	48.390,00	3.268,00	45.122,00	12 (5; 332)	27.585,00	1.211,00	26.374,00	*0,062*
**GF de taxa de materiais por unidade**													
Faturado < Auditado	–21 (–185; –7)	21.000,00	335.711,00	–314.711,00	–21 (–185; –7)	14.668,67	212.657,00	–197.988,00	–21 (–196; –8)	6.331,22	123.054,00	–116.722,00	*0,120*
Faturado > Auditado	15 (5; 33)	46.433,00	1.871,00	44.562,00	17 (5; 37)	34.794,40	915,00	33.879,00	13 (5; 26)	11.638,77	956,00	10.682,00	*0,023*
**GF de órteses, próteses e materiais especiais (OPMEs) por unidade**													
Faturado < Auditado	–172 (–258; –92)	84.649,00	648.804,00	–564.154,00	–142 (–178; –63)	17.938,00	63.800,00	–45.862,00	–247 (–661; –154)	66.710,00	585.002,00	–518.292,00	*< 0,001*
Faturado > Auditado	60 (20; 214)	589.097,00	61.672,00	527.425,00	44 (19; 71)	28.844,00	2.445,00	26.398,00	213 (44; 1024)	560.253,00	59.225,00	501.027,00	*< 0,001*
**GF de taxa de gasoterapia por unidade**													
Faturado < Auditado	–27 (–54; –14)	0,00	668,41	–668,41,00	–27 (–54; xxx)	0,00	109,28	–109,28	–27 (–54; –14)	0,00	559,13	–559,00	*1,000*
Faturado > Auditado	20 (14; xxx)	41,90	0,00	41,90				0	20 (14; xxx)	41,90	0,00	41,00	*0,634*
**Balanço em moeda oficial brasileira (real – R$) para o período das contas faturadas e auditadas, corrigido pelo índice IGP-M (FGV) atualizado^††^**													
Faturado < Auditado				–1.024.692,00				–312.444,00				–711.980,00	
Faturado < Auditado corrigido^††^				–1.344.125,00				–454.895,00				1.036.590,00	
Faturado > Auditado				647.828,00				107.242,00				540.587,00	
Faturado < Auditado corrigido^††^				943.189,00				156.136,00				787.054,00	

*Geral = soma de unidades faturadas, utilizadas em procedimentos diagnósticos e terapêuticos;

^†^md = mediana (1Q = Primeiro quartil e 3Q = Terceiro quartil);

^‡^TotFat = Total faturado;

^II^TotAud = Total auditado;

^¶^Dif = Diferença entre os valores faturados e auditados em moeda oficial brasileira (R$)

**Teste de Mann-Whitney para amostras independentes;

^††^Valores corrigidos pelo índice IGP-M (FGV), considerando período inicial em 07/2020 e período final em 09/2024^(19)^.

A diferença entre mediana Q1 e Q3, denomina-se intervalo interquartil (IQ)^†^. Quanto maiores os IQs entre os grupos sob comparação, maior será a variabilidade dos dados e, consequentemente, menores as chances de detecção de diferenças estatisticamente significativas.

Considerando a faixa “faturado < auditado”, os procedimentos terapêuticos apresentam diferenças significativamente maiores em comparação aos procedimentos de diagnóstico nos grupos sala e OPMEs. Já na faixa “faturado > auditado”, os procedimentos terapêuticos dispõem de diferenças significativamente menores no grupo materiais e maiores no grupo OPMEs (Tabela 5).

## DISCUSSÃO

Neste estudo, o perfil epidemiológico dos beneficiários de OPSs, atendidos no serviço ambulatorial de hemodinâmica, corroborou o de outras pesquisas realizadas em território brasileiro. Nessas pesquisas, preponderaram pacientes do sexo masculino^(20)^, idosos (≥ 60 anos) acometidos por doenças do aparelho circulatório, principalmente as isquêmicas do coração e que demandaram cateterismo cardíaco e angioplastia com *stent* ^(20,21)^, tendo a artéria femoral como a principal via de acesso dos cateteres^(20)^.

Ademais, eram pacientes que apresentaram associação estatística significante para doença arterial coronariana (DAC), com sobrepeso ou obesidade, comorbidades metabólicas prévias e em uso de medicações para controle da hipertensão arterial e do diabetes mellitus^(21)^. Doenças crônicas não transmissíveis (DCNTs) que poderiam ser mais bem controladas nos serviços da atenção primária^(20)^.

O Brasil está entre os países com maior população de idosos^(22)^, a qual triplicará até 2050, passando por uma mudança demográfica acompanhada por transição epidemiológica, caracterizada por multimorbidade de DCNTs^(23)^.

Essa é uma das razões de o Brasil possuir uma das prevalências de multimorbidade mais altas do mundo, de aproximadamente 70%, sendo os idosos os mais acometidos, semelhantemente ao que ocorre em países com altas rendas, como Espanha e Finlândia. Contudo, pode-se considerar o contexto brasileiro de risco, onde a transição epidemiológica tem ocorrido de forma célere e com muitos desafios a serem vencidos^(24)^, em relação às desigualdades sociais e ao custo crescente da assistência à saúde^(25)^.

Por outro lado, os resultados desta pesquisa corroboraram a hipótese de que a ausência da auditoria de enfermagem prévia pode contribuir para maiores perdas financeiras e econômicas pela instituição, especificamente pelo subfaturamento sistêmico de todos os GFs, repercutindo em menor receita financeira, decorrente do serviço de hemodinâmica.

Este estudo sinalizou, as discordâncias referentes às taxas de sala relacionaram-se às cobranças indevidas do tamanho da sala, segundo o porte anestésico do procedimento realizado e autorizado pela OPS, demostrando desconhecimento por parte da equipe administrativa envolvida no processo de faturamento da conta hospitalar.

O subfaturamento no grupo de gasoterapias decorreu da ausência de conferência dos registros assistenciais multidisciplinares, assim como ocorrido no grupo de equipamentos, demostrando desconhecimento ou ausência de conferência pelos colaboradores “não enfermeiros”.

Essas discordâncias exacerbaram-se nos procedimentos terapêuticos relativos aos GFs materiais e medicamentos, sendo considerados de maior retorno financeiro e, portanto, lucrativos, por reunirem itens em maior quantidade e cobrados conforme tabelas praticadas no mercado de saúde suplementar.

O subfaturamento na totalidade dos materiais de consumo se deu pela ausência de registro no prontuário ou na conta contábil do beneficiário, assim como de qualquer ação que implementasse o controle de gastos.

Com relação aos medicamentos, as discordâncias ocorreram basicamente por três motivos: quantidade, cadastro incorreto no sistema e falta de registro em prontuário, que se constituíram nos principais motivos de subfaturamento. Ressalta-se que o cadastro dos itens no sistema segue uma lógica de apresentação do medicamento, sendo que alguns são cadastrados como ampola, outros em miligramas ou mililitros.

Como toda assistência, médica e de enfermagem, gera custo à instituição hospitalar, é relevante que todos esses cuidados assistenciais estejam devidamente descritos e com clareza, para que não haja glosas com prejuízo financeiro, pois os pagamentos se dão mediante os fatos comprobatórios, ou seja, sua descrição, checagem e laudos que comprovem a realização dos procedimentos^(26)^.

Durante a auditoria, encontraram-se dois tipos de discordâncias de cobrança no grupo faturamento OPMEs. A primeira associou-se à divergência da classificação do material, de acordo com a Tabela Simpro^(16)^, de forma que materiais de consumo hospitalar eram cobrados como OPMEs. A segunda discordância apontou que o faturamento de OPMEs balizou-se no valor de compra (pregão) pela instituição, justamente em contas relativas ao convênio de maior demanda de beneficiários, não respeitando a cláusula contratual entre hospital e OPSs de cobrança por cotação junto aos fornecedores cadastrados. Sendo o valor de empenho bem inferior ao valor praticado de cotação, configuraram-se situações que contribuíram para a perda de receita financeira.

Essas ocorrências no processo de autorização e cobrança das OPMEs demonstraram haver desinformação contratual e conceitual dos colaboradores “não enfermeiros”, por serem profissionais sem formação na área da saúde e sem treinamento específico para realização de suas funções.

A presença de um profissional técnico com conhecimento assistencial e administrativo, como o enfermeiro auditor, pode contribuir para que essas lacunas se minimizem. Ademais, a presença desse profissional no processo de aquisição e avaliação desses OPMEs tem garantido a qualidade da assistência prestada e a relação custo-benefício por análise sistemática do prontuário e da auditoria concorrente. Por esses procedimentos é possível detectar inconsistências que, se resolvidas, aumentam a segurança do paciente e reduzem o prejuízo contábil-financeiro para a organização^(27)^.

Estudos recentes evidenciaram que os altos índices de informação incompleta registrada no prontuário do paciente, pela equipe de enfermagem, acarretam graves consequências assistenciais, administrativas e financeiras, sendo que elas poderiam ser minimizadas pela conscientização dos profissionais da saúde por intermédio da auditoria de enfermagem^(28,29)^.

Em estudo reflexivo acerca da auditoria em saúde, como instrumento para qualificar a assistência, também ressaltou que o procedimento traz subsídios à gestão para tomada de decisão, assegurando o equilíbrio financeiro com as melhores práticas clínicas, além de ser um instrumento que permite a implementação de ações para melhoria de processos assistenciais, por meio de ações educativas da equipe^(30)^.

A preocupação na gestão de custos em saúde não é uma prerrogativa de OPSs, o SUS dispõe do Programa Nacional de Gestão de Custos (PNGC) em saúde, criado pelo Ministério da Saúde, como uma importante ferramenta de gestão da rede pública^(31)^.

O PNGC e o Sistema de Apuração e Gestão de Custos do SUS (ApuraSUS) têm como um dos objetivos confrontar os custos estimados dos serviços prestados e seus respectivos recebimentos, assim como os desperdícios gerados pelo uso ineficiente para gerar o produto. A apuração e o controle de custos em saúde permitem a implantação de medidas corretivas para o melhor desempenho das unidades, com base na redefinição das prioridades, no aumento da produtividade e na racionalização do uso de recursos, entre outras medidas administrativas^(31)^.

Dessa forma, as interfaces SUS e Saúde Suplementar são semelhantes no que tange à viabilidade financeira-econômica na prestação da assistência, ao exigirem maior eficiência e menos desperdício na locação dos recursos materiais e financeiros.

O alinhamento da auditoria com as diretrizes e o planejamento estratégico da instituição fortalece a implementação de melhorias e o desenvolvimento de novos recursos capazes de induzir a educação permanecente do corpo clínico e assistencial para o melhor gerenciamento dos recursos existentes, bem como estudos comparativos das melhores formas de remuneração.

Todavia, este estudo contribui para a construção do papel do enfermeiro auditor financeiro, fundamentado em conhecimentos técnicos-científicos e gerenciais para atuar em processos assistenciais e administrativos no mercado de saúde. Trata-se de um profissional executivo, alocado à alta gestão de uma instituição de saúde, com capacidade de subsidiar as decisões tomadas, não somente junto ao serviço de auditoria retrospectiva do faturamento de contas, mas em todo o processo do Núcleo de Saúde Suplementar, como: no credenciamento de novas operadoras; no processo de autorização de procedimentos; na negociação de contratos, tabelas de precificação e pacotes de procedimentos.

Os auditores são necessários para controlar os custos e apoiar os gestores na análise da conta hospitalar, com foco no valor em saúde e não somente no corte de despesas. O controle de custos por auditores visa os resultados para o alcance de assistência integral à saúde dos beneficiários, com a finalidade de maximizar recursos humanos e materiais disponíveis^(8)^.

Para a enfermagem enquanto profissão, o papel emergente do enfermeiro auditor financeiro contribui para o avanço em oportunidades de atuação com autonomia de participação e implementação de decisões para a qualidade do atendimento, em nível de alta gestão da instituição de saúde, raramente ocupada por enfermeiros. Aos cursos de graduação em enfermagem, assim como de pós-graduação *lato* e *stricto sensu* em gerenciamento de enfermagem, recomenda-se a formação de competência econômico-financeira em saúde, para que enfermeiros venham atuar em serviços de saúde públicos e privados, pois não existem processos sem controle de custos.

Essas competências podem reduzir a marginalização do enfermeiro em processos de subordinação de seus cuidados às decisões de outros profissionais, principalmente, quando o setor de saúde sofre pressões e os enfermeiros podem contribuir proativamente, desde aqueles que desempenham um papel clínico até aqueles com funções executivas^(8)^.

A formação da enfermagem para o século XXI exige mudança de paradigmas, de forma que o letramento financeiro faça parte de suas habilidades profissionais, para que o enfermeiro possa contribuir no enfrentamento da sobrecarga econômico-financeira gerada pelo envelhecimento populacional nos sistemas de saúde, em virtude da abrangência obrigatória de procedimentos diagnósticos e terapêuticos.

Consideraram-se como limitações desta pesquisa as relacionadas: (a) à não contemplação direta dos recursos humanos, por estarem indiretamente inseridos na cobrança da taxa de sala; (b) à coleta de dados, em razão da ausência de histórico das autorizações no período de 2019; (c) às restrições metodológicas do desenho retrospectivo, desprovido de grupo controle e possíveis vieses no uso de dados secundários; (d) à não adoção de modelagem econômica formal, por utilizar estatística descritiva e testes não paramétricos.

## CONCLUSÃO

Os resultados da pesquisa corroboraram a hipótese, ao verificarem que as contas hospitalares submetidas à auditoria retrospectiva por enfermeiro com especialização nessa área aumentaram a receita hospitalar, detectando-se subfaturamento em todos os GFs, quando realizados por “não enfermeiros”. Por fim, este estudo contribui para demonstrar a necessidade de formação de enfermeiros com competência econômico-financeira em saúde, vislumbrando a importância do papel emergente do enfermeiro auditor financeiro no mercado da saúde, além de contribuir, por conseguinte, para melhor qualidade da assistência prestada. Ademais, recomenda-se a realização de estudos comparativos mais robustos, para avaliar o impacto da auditoria financeira por enfermeiros, em diferentes contextos hospitalares.

## DISPONIBILIDADE DE DADOS

O banco de dados desta pesquisa estará disponível mediante solicitação ao pesquisador principal, que pode ser contatado por e-mail silvia.meneguin@unesp.br.

# Translation: El desempeño del enfermeiro en la auditoría financiera hospitalaria: un estudio de un caso único

**DOI:** 10.1590/1980-220X-REEUSP-2024-0391es

**Authors:** Silvia Helena Meneguin, Vânia dos Santos Nunes Nogueira, Silvia Cristina Mangini Bocchi

**Affiliations:** 1 Universidade Estadual Paulista, Faculdade de Medicina de Botucatu, Botucatu, SP, Brasil.; 2 Universidade Estadual Paulista, Faculdade de Medicina de Botucatu, Departamento de Clínica Médica, Botucatu, SP, Brasil.; 3 Universidade Estadual Paulista, Faculdade de Medicina de Botucatu, Departamento de Enfermagem, Botucatu, SP, Brasil.

**Keywords:** Rol de la Enfermera, Auditoría de Enfermería, Salud Complementaria, Administración Financiera, Economía y Organizaciones para la Atención de la Salud

## Abstract

**Objetivo:**

Analizar el desempeño del enfermero en la auditoría financiera retrospectiva, en comparación con la realizada por “no enfermeros”, para la remuneración de un hospital por parte de operadores de planes de salud complementarios.

**Método:**

Estudio de caso único, cuantitativo, retrospectivo, documental y analítico, teniendo como unidad de análisis el conjunto de 238 cuentas contables facturadas por “no enfermeros” y auditadas por enfermeros, de procedimientos ambulatorios en el servicio de hemodinámica, en un hospital público del estado de São Paulo, entre 2019 y 2021.

**Resultados:**

Se constató una subfacturación en el rango “facturado < auditado” en el 100 % de los grupos de facturación, independientemente de los procedimientos realizados, con un acumulado de 1 024 692,00 reales, que actualizado hasta septiembre de 2024 correspondería a 1 344 125,00 reales. En los casos de cobro indebido, “facturado > auditado”, hubo un acumulado de 647 828,00 reales.

**Conclusión:**

La auditoría financiera retrospectiva, realizada por enfermeros, aumenta los ingresos hospitalarios. Por último, este estudio contribuye a demostrar la necesidad de formar a estos profesionales con competencias económicas y financieras en el ámbito de la salud, poniendo de relieve la importancia del papel emergente del enfermero auditor financiero en el mercado sanitario.

## INTRODUCCIÓN

El mercado de servicios de salud, ante el riesgo agravado de la insostenibilidad financiera y económica de sus empresas debido al rápido aumento del costo de la asistencia^(1)^, se ha movilizado para gestionar presupuestos reducidos con el fin de equilibrar costos, gastos e ingresos, con el objetivo de garantizar la supervivencia de sus organizaciones.

La escasez de recursos financieros se debe a los avances tecnológicos y a los cambios demográficos, acompañados de una transición epidemiológica hacia la población de edad avanzada^(2)^. De este contexto surge la necesidad de encontrar gestores de servicios de salud capacitados para proporcionar un sistema eficaz de gestión de costos, sin poner en peligro la calidad de los cuidados, con el objetivo de lograr la eficiencia en la distribución de los recursos^(3)^.

Es conocido el hecho que los cuidados de enfermería consumen aproximadamente un tercio del presupuesto hospitalario^(4)^, lo que exige enfermeros con conocimientos y habilidades para tomar decisiones sobre la estructura, los procesos y los resultados institucionales. Por cierto, para la gestión de los servicios de enfermería, es necesario capacitar a las enfermeras en la gestión de costos como una herramienta más a ser utilizada en los procesos de toma de decisiones^(3)^.

En este sentido, la literatura ha corroborado la necesidad de que las y los enfermeras/os que prevén o actúan como gestoras financieras en instituciones de salud alcancen competencia económica y financiera^(5-7)^, para que puedan desarrollar acciones que sustenten los cuidados ofrecidos por estas organizaciones^(8)^ y demostrando que esta es un área de gestión prometedora para profesionales en el rol de auditor^(9)^.

En este estudio, utilizamos la auditoría retrospectiva, operacionalizada después del alta hospitalaria y realizada mediante la verificación sistemática de los cargos por servicios hospitalarios a partir de la historia clínica del paciente^(10)^, para evaluar las distorsiones administrativas y asistenciales en un servicio de hemodinámica.

Existen pocos artículos científicos que relacionen la economía de la salud con la realización de auditorías financieras internas por enfermeros^(8,11,12)^.

Teniendo en cuenta lo anterior, surgió esta pregunta de investigación: ¿Cómo puede contribuir a los ingresos del hospital el trabajo de los enfermeros en las auditorías financieras retrospectivas de las cuentas hospitalarias facturadas por empleados «no enfermeros»?

La hipótesis era que la adopción del proceso de auditoría previa de las cuentas hospitalarias por parte del personal de enfermería aumentaría la eficacia de la gestión de la facturación.

Para comprobar esta hipótesis, el objetivo fue analizar la actuación de los enfermeros en la auditoría financiera retrospectiva, comparada con la realizada por los «no enfermeros», en la remuneración de un hospital por las Operadoras de Planes de Salud (OPS).

## MÉTODO

### Diseño del Estudio

Se trata de un estudio de caso único^(13)^, cuantitativo, retrospectivo, documental y analítico. La unidad de análisis fue la evaluación económica en salud, por lo que se utilizó el guión CHEERS para reportarlo^(14)^.

### Localización y Unidad de Análisis

La unidad de análisis fue un conjunto de 238 cuentas facturadas bajo el modelo de pago *Fee for Service* (cuenta abierta de conceptos de facturación)^(15)^, desde el 01/01/2019 hasta el 31/12/2021, por «no enfermeros» y auditadas por un enfermero. Estas cuentas procedían de beneficiarios de 7 OPS que se sometieron a procedimientos diagnósticos y terapéuticos en el servicio de hemodinámica ambulatoria de un hospital público del estado de São Paulo, Brasil.

La selección de este conjunto derivó de 631 cuentas facturadas, excluyendo 393 que tenían sus beneficiarios hospitalizados o de otros modelos de pago. El análisis se realizó desde la perspectiva del hospital y desde el punto de vista del impacto financiero auditado por la enfermera.

Cabe señalar que el lugar y el período elegidos para este estudio se debieron al hecho de que los procedimientos diagnósticos y terapéuticos son de alto costo, asociados a la falta de control antes de la implementación de la auditoría retrospectiva por parte de las enfermeras en las cuentas contables. Sin embargo, desde el 01/01/2019 hasta el 31/12/2021, estas cuentas fueron facturadas por profesionales administrativos sin formación en el área de la salud, por lo que en este estudio se denominan empleados «no enfermeros».

El servicio de hemodinámica forma parte del Centro de Procedimientos Diagnósticos y Terapéuticos del hospital, con un promedio de 3.700 exámenes mensuales, en su mayoría para beneficiarios del Sistema Único de Salud (SUS), y sólo el 6% de los cuales son para afiliados a la OPS. Los beneficios de este servicio se destinan a mejorar la calidad de la atención prestada por la institución.

### Proceso de Facturación y Auditoría de la Cuenta Contable

La cuenta contable de los servicios prestados por el sector de hemodinámica se desglosa por grupos de facturación (GF), que incluyen: gastos de habitación; gasoterapia; equipos; materiales; medicamentos; órtesis, prótesis y materiales especiales (OPME), así como honorarios médicos.

La tarifación de los precios por día y honorarios sigue los contratos de servicios acordados entre los operadores y el hospital, así como las tablas de referencia en el mercado de la salud, Simpro^(16)^ y Brasíndice^(17)^, para la remuneración de materiales y medicamentos. Para las OPME, sin embargo, se utilizan los valores de cotización del proveedor.

La facturación de la cuenta comienza con la admisión del beneficiario en el servicio, solicitando autorización de las OPS, de acuerdo con las necesidades pre y post procedimiento, y sólo termina con el envío de la factura de pago a los operadores, después de haber sido auditada por una enfermera especializada en esta área.

El objetivo de esta auditoría es revisar la factura de acuerdo con el contrato de servicios acordado con la OPS, los registros de atención multidisciplinaria en la historia clínica electrónica del beneficiario y las autorizaciones pertinentes para todo el proceso de atención. Este procedimiento permite hacer ajustes de acuerdo con la necesidad de incluir o excluir ítems en la factura, para que sea enviada a la OPS con el rigor y la equidad de un servicio de calidad.

El proceso de post-facturación involucra a varios sectores, pues está vinculado al pago y transferencia de honorarios médicos, que siguen al resultado del recurso de descuentos para dar de baja de la facturación del hospital.

### Variables

*Para caracterizar a los beneficiarios de la unidad de análisis:* sexo (F/M); edad por grupos de edad en años (20 a 29, 30 a 39, 40 a 49, 50 a 59 y ≥ 60); diagnósticos médicos en las solicitudes de pruebas (clasificados por grupos y capítulos del Código Internacional de Enfermedades - CIE 10) ^(18)^; clasificación de los procedimientos diagnósticos y terapéuticos (angiografía, cateterismo cardíaco, angioplastia con *stents* cardíacos y cerebrales, reparación quirúrgica interauricular, quimioembolización e implantación transcatéter de prótesis valvulares); vía de acceso para los exámenes (braquial, radial y femoral).

*Independientes*: los tipos de procedimientos, agrupados en terapéuticos y diagnósticos, realizados en el servicio de hemodinámica.

*Dependientes:* resultados entre los facturados por un administrativo «no enfermero» (y no del área de salud) y los auditados por una enfermera entrenada en auditoría, de los ítems clasificados en las franjas de distribución «facturado = auditado», «facturado < auditado» y «facturado > auditado», para esta investigación, en los GF, denominados honorarios de sala, equipos, medicamentos, materiales, OPME y gasoterapia. Se consideró eficiencia del enfermero auditor cuando detectaba partidas en la franja «facturado < auditado», ya que la mayoría de las partidas agrupadas en la franja «facturado > auditado» son desautorizadas.

### Procedimientos Éticos, Recolección y Análisis de Datos

La recolección de datos se inició entre marzo y octubre de 2022, tras la aprobación del proyecto de investigación por el Comité de Ética de la Investigación, CAAE: 55034221.5.0000.5411, dictamen: 5.528.098.

Los datos provinieron de una fuente primaria del Departamento de Gestión de Actividades Académicas (DGAA), a través de un relevamiento en el Sistema MV del Centro de Informática Médica (CIMED), de 238 facturas facturadas a beneficiarios de siete OPS, atendidos desde el 01/01/2019 al 31/12/2021 por el servicio de hemodinamia ambulatoria del hospital. Estas facturas se imprimieron para que todas pudieran ser sometidas a una auditoría financiera retrospectiva por una de las investigadoras, enfermera con posgrado en Auditoría Sanitaria. Esta intervención se basó en los registros multidisciplinares disponibles en la historia clínica electrónica del paciente en el Sistema MV y de conformidad con los acuerdos contractuales vigentes.

Para obtener análisis de las cuentas de forma estandarizada y, por lo tanto, con reducción de sesgos, se elaboró, junto con la enfermera responsable del servicio de hemodinámica, un instrumento de medición de gastos para los procedimientos elegidos para esta investigación. Cabe señalar que el instrumento de medición de los gastos relativos al material fungible hospitalario y a los medicamentos se probó previamente sin necesidad de adaptarlo a una cuenta piloto con los procedimientos diagnósticos y terapéuticos realizados por el servicio. Los OPME fueron calculados de acuerdo con la descripción quirúrgica registrada en la historia clínica electrónica, utilizando cotizaciones de precios en el momento y registros de la Agencia Nacional de Vigilancia Sanitaria (Anvisa) para identificar la clasificación del producto.

Seis GFs componían la factura hospitalaria auditada: tarifa diaria de habitación; tarifa de equipamiento; tarifa de medicamentos; tarifa de materiales; ortesis, prótesis y materiales especiales (OPME); gasoterapia.

Tras auditar la unidad de análisis, los datos se colocaron en Excel en columnas de facturado y auditado para su análisis comparativo.

Esta investigación utilizó los valores de remuneración de los recursos materiales, medicamentos, OPME y tasas de habitación y equipamiento inherentes a los procedimientos realizados en el servicio de hemodinámica.

Los ítems de GF relacionados a la atención y/o procedimientos en la Recolección de datos retrospectiva fueron identificados a partir de un análisis detallado de los registros médicos y de enfermería en la historia clínica electrónica del beneficiario.

### Análisis Estadístico

Se utilizó estadística descriptiva para analizar las variables relativas a la caracterización de los beneficiarios en el conjunto de cuentas contables.

Para analizar las discordancias en las facturas por grupo de facturación, se compararon los procedimientos diagnósticos y terapéuticos en relación con la distribución de los conceptos en los rangos «facturado = auditado», «facturado < auditado» y «facturado > auditado» en los GF, mediante las pruebas no paramétricas de Chi-cuadrado o exacta de Fisher. Además, estos procedimientos se compararon en relación con las diferencias entre los valores facturados y auditados, utilizando la prueba no paramétrica de Mann-Whitney para muestras independientes.

Las diferencias se consideraron estadísticamente significativas si p < 0,05. Los análisis se realizaron con el programa SPSS, versión 21*.*

Se utilizó el Índice General de Precios de Mercado de la Fundación Getúlio Vargas (IGP-M-FGV) para corregir los valores del balance final, considerando el período inicial en julio de 2020 y el período final en septiembre de 2024^(19)^.

## RESULTADOS

### Caracterización de los Beneficiarios de la Unidad de Análisis

La mayoría de los beneficiarios de OPS atendidos entre el 01/01/2019 y el 31/12/2021 en el servicio de consultas externas de hemodinámica e incluidos en la unidad de análisis de este estudio son hombres (60,5%) y de edad ≥ 60 años (64,7%). En las solicitudes médicas predominan los procedimientos de confirmación diagnóstica (66,0%), seguidos de los terapéuticos (34,0%), dirigidos en su mayoría a enfermedades del aparato circulatorio (96,2%), siendo la cardiopatía isquémica el grupo de mayor incidencia (86,1%). Sin embargo, predomina el cateterismo cardiaco (61,8%), seguido de la angioplastia con *stent* cardiaco (29,0%), realizada predominantemente por acceso radial (84,9%) (Tablas 1, 2 y 3).

**Tabla 1:** Beneficiarios atendidos, del 01/01/2019 al 31/12/2021, por planes de saalud complementarios, en el servicio ambulatorio de hemodinámica de un hospital público del estado de São Paulo, según: sexo, edad en años, tipos de procedimientos y vías de acceso - Botucatu, SP, Brasil, 2024.

Sexo (N = 238)	N	%
Femenino	94	39,5
Masculino	144	60,5
**Edad (en años)** (N = 238)		
20 a 29	2	0,8
30 a 39	5	2,1
40 a 49	18	7,6
50 a 59	59	24,8
≥60	154	64,7
**Tipos de procedimientos** (N = 238)		
Cateterismo cardíaco	147	61,8
Angioplastia con *stent* cardíaco	69	29,0
Angiografía	10	4,2
Quimioembolización	5	2,1
Corrección quirúrgica interatrial	3	1,3
Angioplastia con *stent* cardíaco + angioplastia con *stent* cerebral	2	0,8
Implante transcatéter de prótesis valvular aórtica	2	0,8
**Vía de acceso de los exámenes** (N = 238)		
Radial	202	84,9
Femoral	33	13,9
Braquial	2	0,8
Sin especificar	1	0,4

**Tabla 2:** Diagnósticos por grupos y capítulos del Código Internacional de Enfermedades (CIE 10)*, registrados en los pedidos de exámenes de beneficiarios de Operadoras de Planes de Salud complementarios, atendidos del 01/01/2019 al 31/12/2021, en el servicio ambulatorio de hemodinámica de un hospital público del estado de São Paulo. Botucatu, SP, Brasil, 2024 (N = 238).

Grupos	Códigos (CID)*	N	%
Capítulo IX. Enfermedades del aparato circulatorio*		229	96,2
- Enfermedades isquémicas del corazón	I20, I21, I200, I209, I210, I211, I214, I219, I240, I251, I255	205	86,1
- Otras formas de enfermedad del corazón	I352, I500	14	5,9
- Enfermedades cerebrovasculares	I671, I620, I61	8	3,4
- Enfermedades de las arterias, de las arteriolas y de los capilares	I702	2	0,8
Capítulo II. Neoplasias [tumores]*		5	2,1
- Neoplasias malignas de los órganos digestivos	C24, C220	5	2,1
Capítulo XVII. Malformaciones congénitas, deformidades y anomalías cromosómicas*		3	1,3
-Malformaciones congénitas del aparato circulatorio	Q211	3	1,3
Capítulo VI. Enfermedades del Sistema Nervioso*		1	0,4
- Trastornos episódicos y paroxísticos	G459	1	0,4
**Total**		**238**	**100,0**

*OMS, OPAS.

CID-10 - Classificação Estatística Internacional de Doenças e Problemas Relacionados à Saúde. 10ª ed. OMS, OPAS, editores. São Paulo: Edusp; 2017. 1200 p.

**Tabla 3:** Distribución de procedimientos por grupos diagnósticos y terapéuticos de exámenes para beneficiarios de Operadoras de Planes de Salud complementarios, atendidos de 01/01/2019 a 31/12/2021, en el servicio ambulatorio de hemodinámica de un hospital público del estado de São Paulo. Botucatu, SP, Brasil, 2024 (N = 238).

Procedimientos	Diagnósticos		Terapéuticos		Total	
	N	%	N	%	N	%
Cateterismo cardíaco	147	61,8	–	–	147	61,8
Angioplastia con *stent* cardíaco	–	–	69	29,0	69	29,0
Angiografía	10	4,2	–	–	10	4,2
Quimioembolización	–	–	5	2,1	5	2,1
Corrección quirúrgica interatrial	–	–	3	1,3	3	1,3
Angioplastia con *stent* cardíaco + angioplastia con *stent* cerebral	–	–	2	0,8	2	0,8
Implante transcatéter de prótesis valvular aórtica	–	–	2	0,8	2	0,8
**Total**	**157**	**66,0**	**81**	**34,0**	**238**	**100,0**

### Diferencias en las Facturas por Grupo de Facturación

En los grupos de habitación, medicamentos, material, OPMEs y gasoterapia, existe una diferencia estadísticamente significativa entre los procedimientos diagnósticos y terapéuticos en relación con la distribución de los conceptos entre las franjas: «facturado = auditado», «facturado < auditado» y «facturado > auditado». En los grupos de habitaciones, medicamentos, OPMEs y gasoterapia, el rango «facturado < auditado» es más frecuente entre los procedimientos terapéuticos, mientras que este mismo rango es menos frecuente entre los procedimientos terapéuticos del grupo de materiales (Tabla 4).

**Tabla 4:** Comparaciones entre las cuentas contables facturadas por «no enfermeros» y auditadas por enfermeros, según grupos de facturación - GFs (habitación, equipos, medicamentos, materiales, OPMEs y gasoterapia), de beneficiarios de Operadoras de Planes de Salud complementarios, atendidos del 01/01/2019 al 31/12/2021, en un servicio de hemodinámica ambulatoria de un hospital público del estado de São Paulo. Botucatu, SP, Brasil, 2024.

Comparaciones	General(*)		Diagnóstico		Terapéutico		p-valor(**)
	n	%	n	%	n	%	
**Grupo de facturación (GF) tasa de sala por unidad (n = 238)**							
Facturado = Auditado	184	77,3	143	91,1	41	50,6	< 0,001
Facturado < Auditado	42	17,6	8	5,1	34	42,0	
Facturado > Auditado	12	5,0	6	3,8	6	7,4	
**Subtotal**	238	100,0	157	100,0	81	100,0	
**GF tasa de equipamientos por unidad (n = 238)**							
Facturado = Auditado	43	53,8	30	54,5	13	52,0	0,870
Facturado < Auditado	36	45,0	24	43,6	12	48,0	
Facturado > Auditado	1	1,2	1	1,8	0	0,0	
**Subtotal**	80	100,0	55	100,0	25	100,0	
**GF tasa de medicamentos por unidad (n = 238)**							
Facturado = Auditado	316	18,1	180	25,7	136	13,0	< 0,001
Facturado < Auditado	901	51,7	343	48,9	558	53,5	
Facturado > Auditado	527	30,2	178	25,4	349	33,5	
**Subtotal**	1.744	100,0	701	100,0	1.043	100,0	
**GF tasa de materiales por unidad (n = 238)**							
Facturado = Auditado	1.293	21,3	696	17,8	597	27,6	< 0,001
Facturado < Auditado	3.695	60,9	2.460	62,9	1.235	57,1	
Facturado > Auditado	1.083	17,8	753	19,3	330	15,3	
**Subtotal**	6.071	100,0	3.909	100,0	2.162	100,0	
**GF órtesis, prótesis y materiales especiales (OPMEs) por unidad (n = 238)**							
Facturado = Auditado	101	6,5	33	4,4	68	8,5	< 0,001
Facturado < Auditado	734	47,3	334	44,3	400	50,0	
Facturado > Auditado	718	46,2	386	51,3	332	41,5	
**Subtotal**	1.553	100,0	753	100,0	800	100,0	
**GF gasoterapia por unidad (n = 238)**							
Facturado = Auditado	25	55,6	24	88,9	1	5,6	< 0,001
Facturado < Auditado	18	40,0	3	11,1	15	83,3	
Facturado > Auditado	2	4,4	0	0,0	2	11,1	
**Subtotal**	45	100,0	27	100,0	18	100,0	

^(*)^General = suma de unidades facturadas utilizadas en procedimientos diagnósticos y terapéuticos;

(**)Chi-cuadrado o test exacto de Fisher.

La Tabla 5 muestra subfacturación en el rango «facturado < auditado» en el 100% de los GFs, independientemente de los procedimientos diagnósticos o terapéuticos, con un valor acumulado de R$1.024.692,00, que reajustado a los valores hasta septiembre de 2024 correspondería a R$1.344.125,00. Entre los casos de facturación indebida, «facturado > auditado», hay un monto acumulado de R$647.828,00.

**Tabla 5:** Saldo en moneda oficial brasileña (Real - R$), corregido por el índice IGP-M (FGV) de las cuentas contables facturadas por «no enfermeros» y auditadas por enfermeros, por rangos de grupos de facturación - GFs, referentes a procedimientos diagnósticos y terapéuticos realizados a beneficiarios de Operadores de Planes de Salud complementarios, del 01/01/2019 al 31/12/2021, en un servicio de hemodinámica ambulatoria de un hospital público del estado de São Paulo. Botucatu, SP, Brasil, 2024 (N = 238).

Grupos de facturación (GF) por franjas	General en R$*				Procedimientos Diagnósticos en R$				Procedimientos Terapéuticos en R$				p-valor**
	md (1Q; 3Q)†	TotFat‡	TotAud^II^	Dif¶	md (1Q ; 3Q)†	TotFat‡	TotAud^II^	Dif¶	md (1Q ; 3Q)†	TotFat‡	TotAud‡	Dif¶	
**Grupo de facturación (GF) de tasa de sala por unidad**													
Facturado < Auditado	–153 (–300; –144)	22.253,00	31.615,00	–9.362,00	–40 (–142; –33)	3.170,00	3.775,00	–605,00	–153 (–309; –153)	19.352,00	27.840,00	-8.488,00	*< 0,001*
Facturado > Auditado	226 (138; 687)	11.196,00	6.921,00	4.275,00	144 (123; 611)	4.532,00	2.718,00	1814,00	358 (146; 726)	6.664,00	4.203,00	2.461,00	*0,394*
**GF de tasa de equipamientos por unidad**													
Facturado < Auditado	– 28 (–28; –28)	0,00	1.091,00	–1.091,00	– 28 (–28; –28)	0,00	755,00	–755,00	–28 (–28; –28)	0,00	336,00	–336,00	*1,000*
Facturado > Auditado	28 (28; 28)	28,00	0,00	28,00	28 (28; 28)	28,00	0,00	28,00					
**GF de tasa de medicamentos por unidad**													
Facturado < Auditado	–11 (–189; –5)	24.442,00	159.147,00	–134.705,00	–11 (–162; –5)	12.118,00	79.242,00	–67.124,00	–12 (–319; –5)	12.323,00	79.904,00	–67.581,00	*0,320*
Facturado > Auditado	12 (3; 210)	75.975,00	4.480,00	71.495,00	12 (3; 109)	48.390,00	3.268,00	45.122,00	12 (5; 332)	27.585,00	1.211,00	26.374,00	*0,062*
**GF de tasa de materiales por unidad**													
Facturado < Auditado	–21 (–185; –7)	21.000,00	335.711,00	–314.711,00	–21 (–185; –7)	14.668,67	212.657,00	–197.988,00	–21 (–196; –8)	6.331,22	123.054,00	–116.722,00	*0,120*
Facturado > Auditado	15 (5; 33)	46.433,00	1.871,00	44.562,00	17 (5; 37)	34.794,40	915,00	33.879,00	13 (5; 26)	11.638,77	956,00	10.682,00	*0,023*
**GF de órtesis, prótesis y materiales especiales (OPMEs) por unidad**													
Facturado < Auditado	–172 (–258; –92)	84.649,00	648.804,00	–564.154,00	–142 (–178; –63)	17.938,00	63.800,00	–45.862,00	–247 (–661; –154)	66.710,00	585.002,00	–518.292,00	*< 0,001*
Facturado > Auditado	60 (20; 214)	589.097,00	61.672,00	527.425,00	44 (19; 71)	28.844,00	2.445,00	26.398,00	213 (44; 1024)	560.253,00	59.225,00	501.027,00	*< 0,001*
**GF de tasa de gasoterapia por unidad**													
Facturado < Auditado	–27 (–54; –14)	0,00	668,41	–668,41,00	–27 (–54; xxx)	0,00	109,28	–109,28	–27 (–54; –14)	0,00	559,13	–559,00	*1,000*
Facturado > Auditado	20 (14; xxx)	41,90	0,00	41,90				0	20 (14; xxx)	41,90	0,00	41,00	*0,634*
**Balance en moneda oficial brasileña (real – R$) para el período de cuentas facturadas y auditadas, corregido por el índice IGP-M (FGV) actualizado** ^††^													
Facturado < Auditado				–1.024.692,00				–312.444,00				–711.980,00	
Facturado < Auditado corregido^††^				–1.344.125,00				–454.895,00				1.036.590,00	
Facturado > Auditado				647.828,00				107.242,00				540.587,00	
Facturado < Auditado corregido^††^				943.189,00				156.136,00				787.054,00	

*General = suma de unidades facturadas utilizadas en procedimientos diagnósticos y terapéuticos;

^†^md = mediana (1Q = Primer cuartil y 3Q = Tercer cuartil);

^‡^TotFat = Total facturado;

^II^TotAud = Total auditado;

^¶^Diff = Diferencia entre los importes facturados y auditados en moneda oficial brasileña (R$)

**Prueba de Mann-Whitney para muestras independientes;

^††^Valores corregidos por el índice IGP-M (FGV), considerando período inicial en 07/2020 y período final en 09/2024^(19)^.

La diferencia entre las medianas Q1 y Q3 se denomina rango intercuartílico (IQ^†^. Cuanto mayores sean los IQ entre los grupos objeto de comparación, mayor será la variabilidad de los datos y, en consecuencia, menores serán las posibilidades de detectar diferencias estadísticamente significativas.

Considerando el rango «facturado < auditado», los procedimientos terapéuticos presentan diferencias significativamente mayores en comparación con los procedimientos diagnósticos en los grupos de sala y OPME. En el rango «facturado > auditado», los procedimientos terapéuticos presentan diferencias significativamente menores en el grupo de materiales y mayores en el grupo de OPMEs (Tabla 5).

## DISCUSIÓN

En este estudio, el perfil epidemiológico de los beneficiarios de la OPS atendidos en el servicio ambulatorio de hemodinámica corroboró el de otros estudios realizados en Brasil. En estos estudios, hubo preponderancia de pacientes del sexo masculino^(20)^, de edad avanzada (60 años) afectados por enfermedades del sistema circulatorio, especialmente cardiopatías isquémicas que requirieron cateterismo cardíaco y angioplastia con *stent* ^(20,21)^, siendo la arteria femoral la principal vía de acceso para los catéteres^(20)^.

Además, eran pacientes con una asociación estadísticamente significativa con la enfermedad arterial coronaria (EAC), que tenían sobrepeso u obesidad, comorbilidades metabólicas previas y tomaban medicación para controlar la hipertensión y la diabetes mellitus^(21)^. Se trata de enfermedades crónicas no transmisibles (ECNT) que podrían controlarse mejor en los servicios de atención primaria^(20)^.

Brasil se encuentra entre los países con mayor población anciana^(22)^, que se triplicará para 2050, experimentando un cambio demográfico acompañado de una transición epidemiológica, caracterizada por la multimorbilidad de las ECNT^(23)^.

Esta es una de las razones por las que Brasil tiene una de las mayores prevalencias de multimorbilidad del mundo, en torno al 70%, siendo los ancianos los más afectados, de forma similar a lo que ocurre en países de renta alta como España y Finlandia. Sin embargo, podemos considerar de riesgo el contexto brasileño, donde la transición epidemiológica se ha producido rápidamente y con muchos retos que superar^(24)^, en relación con las desigualdades sociales y el aumento del costo de la asistencia sanitaria^(25)^.

Por otro lado, los resultados de este estudio corroboraron la hipótesis de que la ausencia de auditorías previas de enfermería puede contribuir a mayores pérdidas financieras y económicas para la institución, específicamente debido a la subfacturación sistémica de todos los GF, lo que resulta en menores ingresos financieros del servicio de hemodinámica.

Este estudio demostró que los desacuerdos sobre las tarifas de las habitaciones estaban relacionados con el cobro indebido del tamaño de la habitación, de acuerdo con la escala del procedimiento anestésico realizado y autorizado por la OPS, lo que demuestra una falta de conocimiento por parte del equipo administrativo implicado en el proceso de facturación de la factura hospitalaria.

La subfacturación en el grupo de gasoterapia se debió a la falta de comprobación de los registros asistenciales multidisciplinares, al igual que en el grupo de equipos, lo que demuestra un desconocimiento o falta de comprobación por parte del personal «no enfermero».

Estos desacuerdos se agravaron en los procedimientos terapéuticos relativos a los GF de materiales y medicamentos, considerados de mayor rendimiento financiero y, por lo tanto, rentables, ya que contienen artículos en mayor cantidad y se facturan según las tablas practicadas en el mercado complementario de salud.

La subfacturación de todos los consumibles se debió a la ausencia de registro en la historia clínica o en la cuenta contable del beneficiario, así como de cualquier acción de control del gasto.

En cuanto a los medicamentos, los desacuerdos se produjeron básicamente por tres motivos: cantidad, registro incorrecto en el sistema y falta de historia clínica, que fueron las principales causas de la subfacturación. Cabe destacar que el registro de los ítems en el sistema sigue la lógica de la presentación del medicamento, registrándose algunos en ampollas, otros en miligramos o mililitros.

Dado que todos los cuidados médicos y de enfermería generan costos para el hospital, es importante que todos estos cuidados estén correcta y claramente descritos, para que no se produzcan desautorizaciones con pérdidas financieras, ya que los pagos se efectúan sobre la base de hechos justificativos, es decir, su descripción, los controles y los informes que prueban que los procedimientos se llevaron a cabo^(26)^.

Durante la fiscalización, se encontraron dos tipos de desacuerdos de facturación en el grupo de facturación de la OPME. El primero estaba asociado a una divergencia en la clasificación del material, según la tabla Simpro^(16)^, de modo que los consumibles hospitalarios se facturaban como OPME. El segundo desacuerdo fue que la facturación de OPMEs se basó en el valor de la compra (licitación) por parte de la institución, precisamente en las cuentas relativas al convenio con mayor demanda de los beneficiarios, no respetando la cláusula contractual entre el hospital y las OPS de cobrar por cotización de proveedores registrados. Como el monto del compromiso era muy inferior al cotizado, estas situaciones contribuyeron a la pérdida de ingresos financieros.

Estas ocurrencias en el proceso de autorización y cobro de las OPMEs demostraron que los empleados «no enfermeros» carecían de información contractual y conceptual, por tratarse de profesionales sin formación en el área de la salud y sin capacitación específica para el desempeño de sus funciones.

La presencia de un profesional técnico con conocimientos asistenciales y administrativos, como una enfermera auditora, puede ayudar a minimizar estas carencias. Además, la presencia de este profesional en el proceso de adquisición y evaluación de estas OPMEs ha garantizado la calidad de la atención prestada y la relación costo-beneficio mediante el análisis sistemático de las historias clínicas y la auditoría concurrente. Mediante estos procedimientos es posible detectar incoherencias que, si se resuelven, aumentan la seguridad de los pacientes y reducen las pérdidas contables y financieras de la organización^(27)^.

Estudios recientes han demostrado que las elevadas tasas de información incompleta registrada en las historias clínicas de los pacientes por el equipo de enfermería tienen graves consecuencias asistenciales, administrativas y financieras, que podrían minimizarse concienciando a los profesionales de servicios de salud mediante auditorías de enfermería^(28,29)^.

Un estudio reflexivo sobre la auditoría en salud como herramienta de mejora de los cuidados también destacó que el procedimiento ayuda a la gestión en la toma de decisiones, garantizando el equilibrio financiero con las mejores prácticas clínicas, además de ser una herramienta para implementar acciones de mejora de los procesos asistenciales a través de actividades educativas para el equipo^(30)^.

La preocupación con la gestión de costos en salud no es prerrogativa de las OPS; el SUS cuenta con el Programa Nacional de Gestión de Costos en Salud (PNGC), creado por el Ministerio de Salud, como una importante herramienta de gestión de la red pública^(31)^.

Uno de los objetivos del PNGC y del Sistema de Cálculo y Gestión de Costos del SUS (ApuraSUS) es comparar los costos estimados de los servicios prestados y sus respectivos ingresos, así como el desperdicio generado por el uso ineficiente para generar el producto. El cálculo y el control de los costos en salud permiten aplicar medidas correctivas para mejorar el rendimiento de las unidades, basadas en la redefinición de prioridades, el aumento de la productividad y la racionalización del uso de los recursos, entre otras medidas administrativas^(31)^.

De esta forma, el SUS y las interfaces complementarias de salud se asemejan en lo que se refiere a la viabilidad financiera y económica en la prestación de atención, ya que exigen mayor eficiencia y menor desperdicio en la asignación de recursos materiales y financieros.

Alinear la auditoría con las directrices y la planificación estratégica de la institución fortalece la implementación de mejoras y el desarrollo de nuevos recursos capaces de inducir la educación permanente del personal clínico y asistencial para gestionar mejor los recursos existentes, así como estudios comparativos de las mejores formas de remuneración.

Sin embargo, este estudio contribuye a la construcción del rol del enfermero auditor financiero, basado en conocimientos técnico-científicos y gerenciales para actuar en procesos asistenciales y administrativos en el mercado de salud. Se trata de un profesional ejecutivo, asignado a la alta dirección de una institución de salud, con capacidad para apoyar las decisiones tomadas, no sólo en el servicio de auditoría retrospectiva de las cuentas de facturación, sino en todo el proceso del Centro de Salud Suplementario, como por ejemplo: en la acreditación de nuevos operadores; en el proceso de autorización de procedimientos; en la negociación de contratos, tablas de precios y paquetes de procedimientos.

Los auditores son necesarios para controlar los costos y apoyar a los gestores en el análisis de las cuentas del hospital, centrándose en el valor de la asistencia sanitaria y no sólo en la reducción de costos. El control de los costos por parte de los auditores tiene como objetivo obtener resultados en términos de asistencia sanitaria global para los beneficiarios, con el fin de maximizar los recursos humanos y materiales disponibles^(8)^.

Para la enfermería como profesión, el papel emergente de la enfermera auditora financiera contribuye al avance de las oportunidades de participación autónoma y de implementación de decisiones para la calidad de los cuidados, en el nivel de alta dirección de la institución sanitaria, que raramente es ocupado por enfermeras. *Se* recomiendan cursos de pregrado en enfermería, así como cursos de postgrado en gestión de enfermería, para desarrollar *competencias* económicas y financieras en salud, de modo que las enfermeras puedan trabajar en servicios de salud públicos y privados, ya que no hay procesos sin control de costos.

Estas competencias pueden reducir la marginación de las enfermeras en procesos en los que sus cuidados están subordinados a las decisiones de otros profesionales, especialmente cuando el sector de servicios de salud está bajo presión y las enfermeras pueden contribuir de forma proactiva, desde las que desempeñan un papel clínico hasta las que tienen funciones ejecutivas^(8)^.

La formación de enfermería para el siglo XXI requiere un cambio de paradigmas, de modo que la alfabetización financiera forme parte de sus competencias profesionales, para que las enfermeras puedan contribuir a hacer frente a la sobrecarga económica y financiera que genera el envejecimiento de la población en los sistemas de salud, debido al alcance obligatorio de los procedimientos diagnósticos y terapéuticos.

Las limitaciones de esta investigación fueron: (a) no considerar directamente los recursos humanos, ya que están indirectamente involucrados en el cobro de los honorarios por habitación; (b) la recopilación de datos, debido a la falta de autorizaciones históricas en 2019; (c) las restricciones metodológicas del diseño retrospectivo, sin grupo de control y posibles sesgos en el uso de datos secundarios; (d) no adoptar la modelización económica formal, ya que se utilizaron estadísticas descriptivas y pruebas no paramétricas.

## CONCLUSIÓN

Los resultados de la investigación corroboraron la hipótesis, verificando que cuentas hospitalarias sometidas a auditoría retrospectiva por enfermeros especializados en esta área aumentaron los ingresos hospitalarios, detectando subfacturación en todos los GF cuando fueron realizados por «no enfermeros». Por último, este estudio contribuye a demostrar la necesidad de formar enfermeras con competencias económico-financieras en sanidad, destacando la importancia del papel emergente de la enfermera auditora financiera en el mercado de servicios de salud, además de contribuir a una mejor calidad asistencial. Además, se recomienda realizar estudios comparativos más sólidos para evaluar el impacto de la auditoría financiera realizada por enfermeros en diferentes contextos hospitalarios.

## DISPONIBILIDAD DE DATOS

La base de datos de esta investigación estará disponible previa solicitud al investigador principal, con quien se puede contactar por correo electrónico silvia.meneguin@unesp.br.
